# The Transcriptomic Signature of Donkey Ovarian Tissue Revealed by Cross-Species Comparative Analysis at Single-Cell Resolution

**DOI:** 10.3390/ani15121761

**Published:** 2025-06-14

**Authors:** Yu Tian, Yilin Niu, Xinhao Zhang, Tao Wang, Zhe Tian, Xiaoyuan Zhang, Jiachen Guo, Wei Ge, Shuqin Liu, Yujiang Sun, Jianjun Li, Wei Shen, Junjie Wang, Teng Zhang

**Affiliations:** 1 State Key Laboratory of Reproductive Regulation and Breeding of Grassland Livestock (R2BGL), College of Life Sciences, Inner Mongolia University, Hohhot 010070, China; leon.tianyu@hotmail.com (Y.T.); zhangxybio@163.com (X.Z.); 2 College of Animal Science and Technology, Qingdao Agricultural University, Qingdao 266109, China; gewei0901@qau.edu.cn (W.G.); sqliu12@qau.edu.cn (S.L.); s36s@163.com (Y.S.); wshen@qau.edu.cn (W.S.); 3 College of Life Sciences, Qingdao Agricultural University, Qingdao 266109, China; niuyilin25@163.com (Y.N.); xinhaozhang2000@163.com (X.Z.); wangtao990703@163.com (T.W.); tianzhe0609@163.com (Z.T.); 15835447605@163.com (J.G.); 4 National Dezhou Donkey Original Breeding Farm, Binzhou 251903, China; gaizhiqiang197658@163.com

**Keywords:** donkey, ovary, single-cell RNA-seq, cross-species analysis

## Abstract

Donkeys (*Equus asinus*) hold significant agricultural value in China, particularly for their hides and meat, which possess notable medicinal and dietary importance. However, their reproductive efficiency remains suboptimal compared with other livestock. Ovarian function is a key determinant of fertility, yet the molecular mechanisms underlying donkey ovarian biology remain largely unexplored. To address this gap, we performed single-cell RNA sequencing of donkey ovaries, generating a high-resolution transcriptomic atlas comprising 17,423 cells. Cross-species comparative analysis revealed a high degree of evolutionary conservation in core ovarian cell types, including endothelial, epithelial, immune, and smooth muscle cells, among vertebrates. In contrast, granulosa and theca cells exhibited distinct transcriptional profiles across species, reflecting lineage-specific adaptations. Notably, we identified key genes with donkey-specific expression patterns, including *NR3C1* in endothelial cells, *LIPE* in granulosa cells, and *DHRS9* in theca interna cells. Furthermore, an in vitro cumulus–oocyte complex model demonstrated the critical role of *GATM* in mammalian oocyte maturation. Collectively, these findings provide a comprehensive characterization of ovarian cell-type conservation and species-specific adaptations, offering key molecular insights into the mechanisms underlying cross-species differences in reproductive efficiency.

## Simple Summary

This study conducted, for the first time, a single-cell transcriptomic analysis of donkey ovaries and performed a cross-species comparative analysis with the ovaries of zebrafish, mice, macaques, and humans at single-cell resolution. The results revealed a high degree of transcriptional similarity in the endothelial, epithelial, immune, and smooth muscle cells across the species, whereas the granulosa and theca cells exhibited distinct transcriptional atlas.

## 1. Introduction

Donkeys are equids, classified within the order of odd-toed ungulates. As one of the earliest species domesticated by humans, it has considerable economic significance [1], currently attributable to the intrinsic nutritional richness and medicinal value of donkey meat, skin, and dairy [2,3,4]. However, compared with other domesticated species, donkeys exhibit lower reproductive efficiency, highlighting the importance of investigating the underlying biological mechanisms governing their fertility [5].

The ovary is the principal reproductive organ in female fauna and ultimately determines reproductive prowess. Folliculogenesis is initiated by the activation of quiescent primordial follicles and progresses through sequential stages, encompassing the primary, secondary, antral, and preovulatory developmental stages, ultimately culminating in follicle rupture and the release of a mature oocyte [6]. This intricate process requires the meticulous regulation of various cellular events, including the proliferation and differentiation of granulosa cells, the formation of the theca cell layer, and the vascularization of follicles [7,8]. With the advancement of single-cell RNA sequencing (scRNA-seq) technology, a granular understanding of the functional attributes of diverse cell types during follicle development has been clarified at the cellular level. This is reflected in the specifically expressed gene sets, regulatory networks of transcription factors, transcriptomic maps, and interaction networks of different ovarian cell subpopulations [9]. Nonetheless, the molecular characterization at the single-cell level of donkey ovaries remains an area ripe for exploration.

Furthermore, notable discrepancies exist in the mechanisms governing ovarian function across various species. On the one hand, variations in ovarian morphogenesis among species contribute to the species-specific nature of ovarian function. Throughout the evolution of vertebrate ovaries, fish have uniquely preserved germline stem cells enveloped by bipotential supporting cells, which give the majority of female fish the capacity for continuous germ cell production throughout their lifespan. The adult zebrafish ovary exhibits a lobular architecture, comprising multiple distinct ovarian lobules. Zebrafish folliculogenesis progresses through five distinct developmental stages, with the following distribution: primary growth stage (75%), cortical alveolar stage (12%), early vitellogenic stage (10%), late vitellogenic stage (1%), and preovulatory stage (2%) [10]. In mammals, the majority of current research indicates that ovarian follicles undergo a gradual process of development, maturation, and ovulation until they are ultimately depleted and difficult to regenerate [11]. The fundamental structure of the mammalian ovary consists of a central medulla and a peripheral cortex containing oocytes. The ovarian architecture of humans and macaques is similar, with primordial, primary, and secondary follicles constituting approximately 88%, 10%, and 2% of adult human ovarian follicles, respectively [12]. In adult macaques, these proportions are approximately 75%, 15%, and 10%, respectively [13]. Mice exhibit a relatively high follicular density, with primordial, primary, secondary, and antral follicles comprising approximately 55%, 15%, 13%, and 17% of the ovarian follicle population, respectively [14]. In the donkey, the cortical region housing the follicles is situated centrally within the ovary, while its connective tissue and blood vessels are positioned in the outer layer. Consequently, ovulation is confined to a distinct region known as the ovulation fossa. Statistical analyses indicate that in adult donkeys, the primordial, primary, secondary, and antral follicles constitute approximately 91.3%, 8.2%, 0.4%, and 0.1% of the ovarian follicle population, respectively [15]. On the other hand, the regulatory pattern of follicle development has been found to be species-specific. In the case of mammals, there are clear differences between animals that deliver multiple or single births. In singleton animals, follicular development typically exhibits distinct wave-like patterns, wherein one wave of follicles undergoes cyclic recruitment, selection, and dominance, with the subsequent wave emerging shortly before ovulation. Conversely, in multifetal animals, follicles exhibit continuous growth and degeneration throughout the estrous cycle, with less apparent follicular dominance [16]. Despite researchers summarizing the functional characteristics of ovaries in different species, a comprehensive study on the species-specific and molecular characterization of ovaries is lacking to date.

In this study, we successfully constructed the first scRNA atlas of the donkey ovary. Furthermore, we incorporated scRNA-seq datasets from zebrafish, mouse, donkey, macaque, and human ovaries to provide a comprehensive characterization of the conservation of ovarian cell types during long-term evolution. Our findings have elucidated functional gene targets marking distinct cell type characteristics within donkey ovaries, as compared with other species. These results provide key molecular insights into the potential mechanisms underlying the cross-species differences in reproductive efficiency.

## 2. Materials and Methods

### 2.1. Ethics Statement

The total operating steps of donkey experiments were approved by the requirements of the Animal Ethics Committee of Qingdao Agricultural University (approval No. 2021-011). The mouse experiments were also reviewed and approved by the Animal Ethics Committee of Qingdao Agricultural University (approval No. 2023-021).

### 2.2. Experimental Animals

This study used Dezhou donkeys (Wutou strain) about 2 years old, which were provided by the national Dezhou donkey original breeding farm in Binzhou, Shandong Province, China. Upon slaughter, after removal of adipose, blood, and impurities, the ovarian tissue was promptly collected after washing in PBS and stored on ice. To ensure experimental consistency, all slaughtered donkeys were in estrus. Estrus status was determined through a combination of behavioral and external signs, supplemented by transrectal ultrasonographic examination using an RKU10 fully digital B-ultrasound diagnostic system equipped with a 6.5 MHz rectal linear probe. The probe was inserted into the rectum to the level of the pelvic inlet and then maneuvered along the uterine body and horns at an angle of 45° to 90°. Continuous adjustments were made to obtain clear images of the ovaries and follicles. Follicular measurements revealed that the dominant follicle measured 25.9 ± 3.7 mm in diameter, while the preovulatory follicle measured 36.81 ± 3.78 mm in diameter. All ovaries included in this study contained both dominant and preovulatory follicles and lacked a corpus luteum, thereby confirming their suitability for subsequent analyses. In this study, scRNA-seq was performed on ovarian tissue from one donkey to generate a comprehensive transcriptomic atlas. To validate the principal findings and ensure experimental reproducibility, immunofluorescence analyses were performed using ovarian tissues from three independent donkeys (*n* = 3).

The C57/BL6 mice utilized in the experiments were procured from Vital River Laboratory Animal Technology Co., Ltd. (Beijing, China). They were housed in controlled environmental conditions, maintained at temperatures between 20 and 22 °C, with ad libitum access to standard chow and water. Ovarian tissue was harvested from mice at the age of 2 months for experiments. The number of follicles in the ovaries of the mice used in this study were quantified as follows: primordial follicles, 91.1 ± 11.59; primary follicles, 22.8 ± 6.36; secondary follicles, 21.8 ± 5.43; and antral follicles, 28.7 ± 6.15.

All the applicable institutional and national guidelines for the care and welfare of animals have been strictly followed for the tissue sampling procedures.

### 2.3. Single Cell Suspension Preparation and Sequencing

Given the substantial size of donkey ovaries, we adopted the ovarian tissue preparation methods for single cell sequencing as described by Pei et al. and Han et al. [17,18]. Specifically, the ovary was sectioned into five regions along its long axis, and each region was further minced into approximately 5 mm cubed pieces. From these, 2 cortical pieces were randomly selected per region, yielding a total of 10 ovarian cortical pieces for processing. The isolated ovarian tissue was then dissected into smaller fragments using a sterile, enzyme-free scalpel and subjected to enzymatic digestion in a solution containing 0.25% trypsin (Hyclone, Beijing, China) and collagenase (2 mg/mL, Sigma-Aldrich, C5138, Shanghai, China) for 15 min at 37 °C. The enzymatic digestion was halted by adding 10% fetal bovine serum (FBS). Subsequently, the digested tissue was mechanically dissociated using a pipette and filtered through a 100 μm cell strainer. Finally, the collected cell suspension was washed three times with PBS containing 0.04% bovine serum albumin (BSA) to obtain a purified cell suspension. In accordance with the DNBelab C Series single-cell library preparation protocol, the cell suspensions underwent droplet generation, emulsion breakage, bead collection, reverse transcription, and cDNA amplification to produce barcoded libraries [19]. Subsequent sequencing was conducted utilizing a DNBSEQ-T7 sequencer (Shenzhen, China).

### 2.4. The Quality Control and Preprocessing of scRNA-Seq Data

The reference genome was downloaded from the Ensembl database (assembly: ASM1607732v2). The raw data underwent preprocessing using DNBC4tools (v2.3.0) to generate single-cell gene expression matrices and barcode matrix. Subsequently, the single-cell gene expression matrix was read using Seurat software (v4.3.0), while SoupX (v1.6.2) was employed to eliminate environmental RNA contamination, and DoubletFinder (v2.0.3) was utilized to remove doublet contamination. Furthermore, to ensure the retention of high-quality cells, cells with fewer than 200 genes and more than 10% mitochondrial gene count were removed, and the genes expressing in fewer than 3 cells were filtered out.

The integrated analysis of multiple samples of donkey ovaries was conducted using the “FindIntegrationAnchors” and “IntegrateData” functions in Seurat. Subsequently, the data from different replicates underwent normalization using the “NormalizeData” function. Following normalization, the most variable genes were determined using the “FindVariableFeatures” function with the “vst” method.

### 2.5. Transcriptional Similarity Analysis Between Cell Types Across Species

We collected scRNA-seq datasets from human (https://www.ncbi.nlm.nih.gov/geo/query/acc.cgi?acc=GSE118127 (accessed on 19 August 2023)), macaque (https://db.cngb.org/search/project/CNP0001469/ (accessed on 19 August 2023)), mouse (https://www.ncbi.nlm.nih.gov/geo/query/acc.cgi?acc=GSE198832 (accessed on 19 August 2023)), and zebrafish (https://www.ncbi.nlm.nih.gov/geo/query/acc.cgi?acc=GSE130487 (accessed on 19 August 2023)) ovaries from publicly available databases.

Using the Ensembl Biomart tool, the orthologous genes among humans, macaques, donkeys, mice and zebrafish were identified. Employing human as the reference species, genes annotated as “one2one_ortholog” in other species were retained [20], resulting in a total of 11,085 orthologous genes. These genes were subsequently selected for comprehensive comparative and functional analyses. The cell count matrix of orthologous genes were extracted from the scRNA-seq data of human [21], macaque [22], donkey, mouse [23] and zebrafish [24]. Cross-species scRNA-seq data integration was performed using the reciprocal principal component analysis (RPCA) method. Subsequently, the MetaNeighbor software (v1.20.0) was employed to evaluate transcriptional similarities between cell types across species. The area under the receiver operator characteristic curve (AUROC) score was utilized to quantify the similarity of cell types in pairs [25]. The “FindAllMarkers” function was used to identify cell type-specific marker genes for distinct cell populations within the ovary.

### 2.6. Multispecies Correlation Analysis

The scRNA-seq data from donkey, human, macaque, mouse, and zebrafish were integrated. Following data normalization using the NormalizeData function in Seurat, the top 2000 most variable genes for each replicate were determined using the “vst” method via “FindVariableFeatures” function. Subsequently, Spearman correlations were computed based on the average expression values of this gene set across the specific cell type in different species.

### 2.7. Gene Regulatory Network Inference

Following the construction process outlined by SCENIC for the cisTarget database (https://github.com/aertslab/create_cisTarget_databases (accessed on 28 August 2023)), we generated cisTarget databases tailored to donkey, macaque, and zebrafish. The construction method of the “tbl” file follows the protocol outlined by JoGraesslin’s script (https://github.com/JoGraesslin/Zebrafish_SCENIC (accessed on 28 August 2023)). Specifically, the orthologous gene table for humans and other species, including Ensembl ID and gene symbol, was retrieved from the Ensembl database (https://www.ensembl.org/biomart/martview/ (accessed on 28 August 2023)). Next, the human TF annotation file from the SCENIC database (https://resources.aertslab.org/cistarget/motif_collections/v10nr_clust_public/snapshots/motifs-v10-nr.hgnc-m0.00001-o0.0.tbl (accessed on 28 August 2023)) was imported. The human TF annotation file was then merged with the orthologous gene table, and duplicate entries were removed. Finally, the human gene annotations were updated to their corresponding orthologs in other species.

We employed pySCENIC to infer ovarian cell-specific gene regulatory networks. Initially, the gene regulatory networks were inferred utilizing the GRBboost2 algorithm, while the cis-regulatory motif analysis was conducted on each co-expressed module using the “pyscenic ctx” function. Following this, the target genes of the transcription factors underwent scoring by AUCell, and the regulator activity was calculated accordingly [26]. Additionally, the “calcRSS” function was utilized to identify transcription factors specific to each single-cell subpopulation. The regulon specificity score (RSS) was defined based on the Jensen–Shannon divergence [27]. Transcription factors were considered significant when their AUCell score > 0.1 and Z-score normalized regulon specificity score (RSSZ) > 1.0.

To identify transcription factor modules, we employed the pipeline proposed by Suo et al. Firstly, the Pearson correlation coefficient (PCC) of each pair of transcription factors was computed based on the transcription factor activity scores of each cell. For a given pair of regulons A and B, the corresponding connection similarity index (CSI) was defined as the fraction of regulons whose PCC with both A and B was lower than the PCC between A and B. Finally, we extracted the data matrix with CSI values exceeding 0.8 and applied the Ward clustering method to identify transcription factor modules [27].

### 2.8. Analysis of Differentially Expressed Genes in Cell Clusters

We utilized the “FindMarkers” function to conduct a comparison of differential gene expression between two specific cell clusters, employing the non-parametric Wilcoxon rank sum test. Genes meeting the criteria of an adjusted *p*-value < 0.05 (corrected using the Bonferroni method) and |log_2_FC| > 0.25 were identified as significantly differentially expressed genes.

### 2.9. Protein–Protein Interaction Analysis

We utilized the String database (https://www.string-db.org (accessed on 19 November 2023)) to retrieve protein–protein interaction data, and subsequently employed Cytoscape software (v3.9.1) for constructing a cell interaction network.

### 2.10. Evaluation of Metabolic Activity

The Single-Cell Metabolic Landscape pipeline (https://github.com/LocasaleLab/Single-Cell-Metabolic-Landscape (accessed on 6 December 2024)) was employed to evaluate metabolic pathway activities in granulosa cells across different species. Initially, the mean expression level of each metabolic gene in granulosa cells was determined. Metabolic genes were obtained from the KEGG database (http://www.kegg.jp (accessed on 6 December 2024)). Subsequently, the expression level of each gene within a specific cell type was normalized by comparison to its mean expression level across all cell types, yielding a relative expression metric. For each metabolic pathway, the weighted average expression of all associated genes was calculated. To minimize the impact of genes with low expression or high deletion rates, outliers with relative expression levels greater than three times the 75th percentile or less than one-third of the 25th percentile within each pathway were excluded. A random permutation test was employed to evaluate the statistical significance of pathway activity in each cell type. The cell type labels were randomly shuffled 5000 times to simulate the null distribution of pathway activity scores, which were then compared with the pathway activity scores from the original dataset. *p*-values were calculated to determine whether the pathway activity in each cell type significantly deviated from the average [28].

Furthermore, scFEA was applied to infer cellular metabolic fluxes from scRNA-seq data [29]. After generating the granulosa cell count matrix, the script “scFEA.py” was utilized to compute the metabolic flux profiles and relative abundances of granulosa cells across different species. To quantify interspecies differences in metabolic fluxes, Cohen’s D function was used to assess the effect size of flux variations.

### 2.11. Single-Cell Developmental Trajectory Construction

Monocle software (v2.24.0) was employed to infer cell developmental trajectories. Initially, the cell gene count matrix and cell annotation data were extracted from the Seurat object and a Monocle analysis object was created using the “newCellDataSet” function. Dimensionality reduction and cell ordering were then conducted using the “DDRTree” method and “orderCells” function. Finally, the “BEAM” function was employed to identify differential genes along the pseudotime developmental trajectory [30].

### 2.12. Transcriptional Regulatory Network Analysis

To identify the potential key transcriptional regulators within the gene set, we performed regulatory network analysis using the R package GENIE3 (v1.22.0) [31], based on UMI-normalized expression data. Species-specific upregulated genes in theca cells of different species were used as input for the network analysis.

### 2.13. Cell–Cell Interaction Analysis

The CellChat software (v1.6.1) was applied to elucidate the interaction network among ovarian cell populations across different species. As described in the pipeline, the input file for CellChat was generated using the single-cell gene count matrix produced by Seurat, while the “computeCommunProb” and “aggregateNet” functions were applied to compute the frequency and intensity of cell interactions across various cell types. To mitigate the influence of cell proportions on the calculation results, we utilized the “population.size” parameter [32].

NicheNet software (v2.0.5) was employed to uncover the distinct ligand signals received by granulosa cells in the ovaries of donkey compared with those received by granulosa cell in various species [33]. The analysis process was followed the guidelines outlined in the Github vignette (https://github.com/saeyslab/nichenetr/tree/master/vignettes (accessed on 19 December 2023)). Granulosa cells were designated as receptor cells, while endothelial cells, immune cells, smooth muscle cells, and theca cells were designated as sender cells.

### 2.14. GO and KEGG Enrichment Analysis

In the study, the clusterProfiler software (v4.8.3) and Metascape were utilized to conduct Gene Ontology (GO) and Kyoto Encyclopedia of Genes and Genomes (KEGG) enrichment analyses on the designated gene set [34,35].

### 2.15. Immunofluorescence Staining

Ovarian tissue samples from donkeys and mice were fixed in 4% paraformaldehyde at 4 °C overnight, transferred to 70% ethanol for storage, and subsequently embedded in paraffin. The paraffin-embedded tissues were sectioned at a thickness of 5 μm. The sections were deparaffinized with xylene and rehydrated through a graded ethanol series (100%, 90%, 80%, and 70%), followed by rinsing with distilled water. Antigen retrieval was performed by incubating the sections in 0.01 M sodium citrate buffer (pH 6.0) at 96 °C for 15 min in a water bath, followed by gradual cooling to room temperature. To block nonspecific binding, sections were incubated with 5% BSA for 1 h at room temperature. Subsequently, primary antibodies were diluted in blocking buffer and applied to the sections, followed by overnight incubation at 4 °C (Table S1). The following day, the sections were washed three times with PBS (5 min each) and incubated with the appropriate secondary antibodies for 1 h at 37 °C in the dark (Table S1). After an additional three washes with PBS, the nuclei were counterstained with DAPI (Bioss, C02-04002, Beijing, China) for 10 min. Finally, the sections were mounted with antifade mounting medium and imaged using a fluorescence microscope (Olympus, BX51, Tokyo, Japan).

The specificity of the antibody for its intended target was validated through sequence homology analysis and negative control experiments. In the negative control experiments, the primary antibody was omitted, and samples were incubated solely with the secondary antibody.

### 2.16. Cumulus–Oocyte Complex (COC) Collection and Small Interfering RNA (siRNA) Transfection

Female mice aged 7–8 weeks were injected with 5 IU of pregnant mare serum gonadotropin (PMSG). After 48 h, COCs were harvested by disrupting ovarian follicles. The selected ovaries exhibited multiple prominent follicles on their surface, characterized by intact and evenly distributed blood vessels, as well as transparent follicular contents. Antral follicles visible on the ovarian surface were carefully punctured, and COCs were isolated. Only COCs with more than three layers of cumulus cells, an intact structure, and a uniform oocyte cytoplasm were selected for further analysis. These COCs were cultured in IVM medium, which consisted of M199 medium, 0.57 nmol/L l-cysteine, 0.5 IU/mL FSH, 0.5 IU/mL LH, 10 ng/mL EGF, and 1% (*v*/*v*) penicillin and streptomycin.

We synthesized siRNA (siGatm) and its negative control (NC) at GenePharma (Table S2). Following the method of Zhang et al. [36], siRNA was mixed with GP-transfect-Mate (GenePharma, Suzhou, China) and added to IVM medium to achieve a final transfection concentration of 100 nM. A total of 20–30 COCs were placed in 100 μL of this medium and incubated for 24 h. Subsequently, the COCs were digested with 0.1% hyaluronidase to separate the oocytes from the cumulus cells.

### 2.17. Detection of Spindle Assembly

The mice used in this experiment were 7–8 weeks old. Oocytes were fixed in 4% paraformaldehyde (PFA) for 30 min and then permeabilized with 0.5% Triton X-100 for 20 min. Following permeabilization, the oocytes were blocked in 1% BSA for 1 h. The oocytes were then stained with an anti-α-Tubulin FITC antibody (1:500, T6199, Sigma-Aldrich, MO, USA) and incubated overnight at 4 °C. The following day, unbound dye was removed using 0.1% PVA-PBS. Finally, the oocytes were mounted with antifade mounting medium Hoechst 33342. Spindle/chromosome structures were analyzed using a Laser Scanning Confocal Microscope (Leica TCS SP5 II, Wentzler, Germany). Each group consisted of 20–30 oocytes, with five independent biological replicates conducted to ensure reliability and reproducibility of the results.

### 2.18. Reactive Oxygen Species (ROS) and Mitochondrial Membrane Potential Evaluation

ROS and mitochondrial membrane potential levels in oocytes were measured using the Reactive oxygen species assay kit (Beyotime, S0033S, Shanghai, China) and the Mitochondrial membrane potential assay kit (Beyotime, C2006), respectively. Oocytes were transferred to DCFH-DA (1:1000) and JC-1 (1:1000) detection solutions and incubated at 37 °C in an environment with 5% CO_2_ for 20 min. Fluorescence images were then captured using a fluorescence microscope (Olympus, APX100), and the mean fluorescence intensity of the oocytes was calculated using ImageJ software (v1.48, National Institutes of Health, Bethesda, MD, USA). Each group comprised 20–30 oocytes and was conducted with five independent biological replicates to ensure robust and reliable results.

### 2.19. RNA Extraction and Real-Time Quantitative PCR (RT-qPCR)

RNA was extracted from cumulus cells collected from 50 COCs using the SPARKeasy RNA extraction kit (Sparkjade, AC0202, Qingdao, China). Reverse transcription was then carried out using SPARKscript II RT Plus (Sparkjade, AG0304). Finally, RT-qPCR was conducted using SYBR Premix Ex Taq™ fluorescent dye and a CFX96 Real-Time PCR instrument (BioRad, CFX96, Hercules, CA, USA). The experiment was conducted with five independent biological replicates to enhance the accuracy and reproducibility of the findings. The sequences of the primers used in this study are listed in Table S3.

### 2.20. Phylogenetic Trees

Phylogenetic trees and divergence times were inferred using the TimeTree database (http://www.timetree.org/ (accessed on 20 August 2023)) [37].

### 2.21. Statistical Analysis

Statistical analyses were conducted using GraphPad Prism 8 software. Bioassay data are presented as mean ± SD. Data were analyzed using either *t* test or one-way ANOVA.

## 3. Results

### 3.1. Identification of Ovarian Cell Types in Dezhou Donkey

Using scRNA-seq, firstly, we conducted a comprehensive and unbiased classification of ovarian cells from the Dezhou donkey. Following stringent quality filtering procedures, a total of 17,423 high-quality transcript profiles representing ovarian cells (Ovary1: 6149, Ovary2: 5192, Ovary3: 6082) were acquired (Figure S1A,B). Furthermore, 16 distinct cell clusters were generated using uniform manifold approximation and projection (UMAP) analysis (Figure S1C). To elucidate the diversity of ovarian cell types, we identified the signature genes associated with each cluster. Additionally, five cell types were identified in the donkey ovary, comprising endothelial cells (*VWF*, clusters 0, 1, 2, 3, 5, 6, and 11) [21], granulosa cells (*FSHR* and *NR5A2,* cluster 9) [22], T cells (*CD3E*, cluster 10) [38], neutrophils (*CXCL8*, clusters 12 and 15) [38], smooth muscle cells (*ITGA7*, clusters 4, 7, 8, and 14) [39], and theca cells (*MFAP4* and *CYP17A1*, cluster 13) [22] (Figure S1D,E). Following cell type identification, we conducted GO enrichment analysis with genes highly expressed in each specific cell type, unveiling distinct biological functions associated with each. Notably, the granulosa cells exhibited enrichment in the biological functions related to the cell cycle process and energy metabolism, while theca cells displayed specific upregulation of the genes involved in amino acid metabolism (Figure S1F).

### 3.2. Cross-Species Ovarian Cell Transcriptional Similarity Analysis

**Figure 1:**
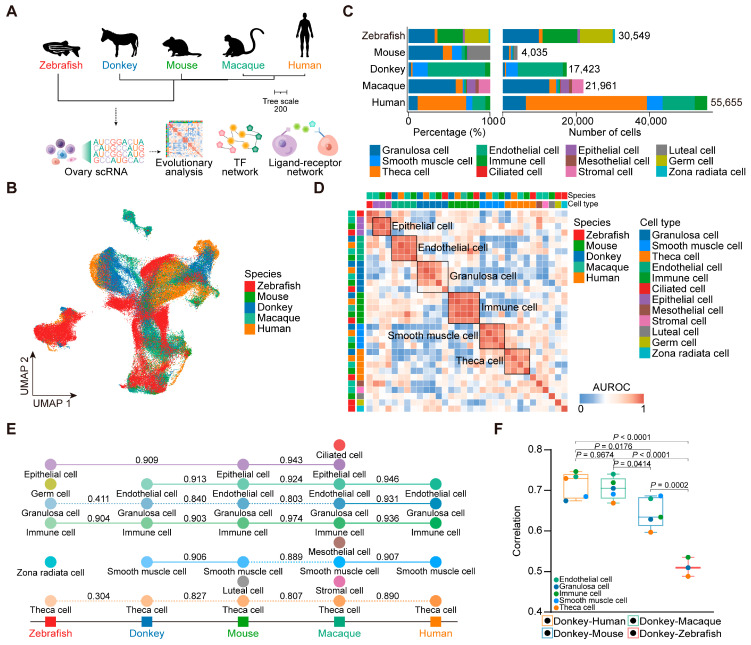
Integrated analysis of ovary scRNA-seq data from five species. (**A**) Overall experimental design. (**B**) UMAP plot of ovarian cells colored by species. (**C**) Bar chart showing number and percentage of cell types for each species. (**D**) Correlation of orthologous gene expression between zebrafish, mouse, donkey, macaque, and human cell types based on AUROC scores. (**E**) A dendrogram arranged according to the evolution of animals from lower organisms to higher organisms, with the AUROC scores of neighboring species marked. (**F**) Boxplot of Spearman’s correlation of mean gene expression between two different species. Each dot represents a cell type.

This study integrated scRNA-seq data from diverse species, including the zebrafish [24], mouse [23], donkey, macaque [22], and human [21] (Figure 1A). Consistent with the aforementioned quality control and annotation procedures, we analyzed a total of 129,623 cells and classified them into 12 distinct cell types across the ovaries of various species (Figure 1B and Figures S2–S5). Specifically, zebrafish displayed six ovarian cell types, totaling 30,549 cells; mouse, seven ovarian cell types, totaling 4035 cells; macaque, nine ovarian cell types, totaling 21,961 cells; and human, five ovarian cell types, with a total of 55,655 cells (Figure 1C).

To evaluate the similarity of ovarian cell populations across the species, we applied MetaNeighbor analysis [40]. The AUROC score heatmap of ovarian cells across the five species revealed notable patterns. Specifically, immune cells exhibited robust cross-species similarities among vertebrates, with conserved gene expression profiles observed in granulosa cells, endothelial cells, and smooth muscle cells across mammals (Figure 1D). Furthermore, we explored the conservation of ovarian cells in neighboring species during evolution based on a phylogenetic tree. As expected, macaque and human ovarian cells displayed a high degree of conservation. Interestingly, the ovarian epithelial cells of zebrafish and mice, and mice and macaques showed a high degree of conservation (Figure 1E). Subsequently, we examined the transcriptome dynamics in the same cell types between donkeys and the other vertebrates. Overall, the correlation between donkeys and humans, as well as between donkeys and macaques, was notably higher compared with that between donkeys and mice, as well as between donkeys and zebrafish. At the cellular level, theca cells exhibited the lowest correlation between donkeys and macaques, and donkeys and mice, as well as donkeys and zebrafish, meanwhile a stronger correlation was observed between donkeys and humans. Additionally, granulosa cells displayed a lower correlation between donkeys and the other vertebrates (Figure 1F). Overall, these findings underscore the conservation of immune cells, endothelial cells, epithelial cells, and smooth muscle cells across the vertebrate ovaries, while also highlighting the unique transcriptional signatures of granulosa cells and theca cells among the species.

### 3.3. Conserved Transcription Factor Modules in Ovarian Cells Across Species

**Figure 2:**
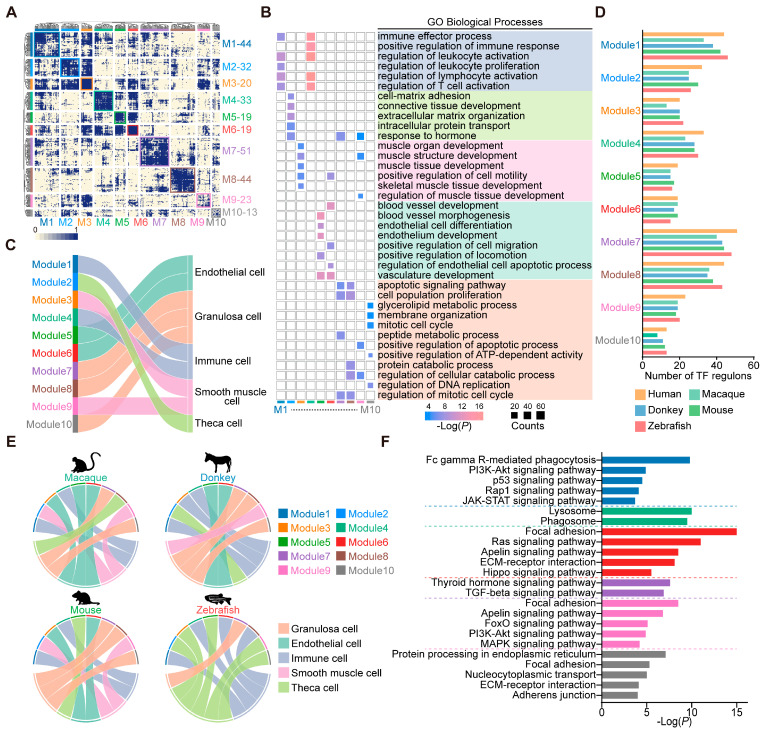
Transcription factor regulatory programs in vertebrate ovary cell-type evolution. (**A**) Identification of 10 human transcription factor modules based on the regulon connection specificity index (CSI) of the human ovary cell landscape. (**B**) GO analysis of target genes of transcription factors. (**C**) Sankey plot showing the connectivity between transcription factor modules and human ovary cell categories. (**D**) Bar chart showing the number of transcription factors in each module in 5 vertebrates. (**E**) Circos plots showing connectivity between transcription factor modules and cell categories in macaque, donkey, mouse, and zebrafish ovaries. (**F**) Enriched KEGG signaling pathways of orthologous target genes regulated by modules 1, 4, 6, 7, 9, and 10.

Transcription factors play a central role in maintaining cell identity and often cooperate to coordinate cellular gene expression levels [27]. We employed the SCENIC methodology to discern the transcription factors exhibiting high regulatory activity within human ovarian cells; subsequently 10 distinct transcription factor modules based on CSI were identified (Figure 2A). We then conducted GO enrichment analysis on the target genes of transcription factors across various modules (Figure 2B). Among them, modules 1 and 4 exhibited a pronounced enrichment in immune-related biological processes, suggesting a prominent role in immunological regulation. Module 2 manifested enrichments in functions like “cell–matrix adhesion” and “response to hormone”. Modules 3 and 9 demonstrated enrichment in biological processes pertinent to muscle development. Modules 5 and 6 were associated with endothelial cell development, as evidenced by the enrichment of relevant biological functions. Furthermore, modules 7, 8, and 10 collectively participated in biological processes primarily linked to cell cycle regulation and energy metabolism (Figure 2B). Based on the primary biological functions ascribed to the regulation of transcription factors within each module, we assigned modules to relevant cell types to reflect their regulatory specificity (Figure 2C). For instance, modules 1 and 4 exhibited a regulatory influence predominantly on immune cells. Module 2 specifically governed theca cells, while modules 3 and 9 exhibited regulatory specificity for smooth muscle cells. Modules 5 and 6 were intricately involved in regulating endothelial cells. Meanwhile, modules 7, 8, and 10 participated in the regulatory mechanisms governing granulosa cells.

To demonstrate the conservation of transcription factor modules in ovarian cells, we employed the switching of orthologous transcription factors between humans and the other vertebrates. A total of 231 orthologous transcription factors were identified in the macaques, 251 in donkeys, 268 in mice, and 279 in zebrafish. These orthologous transcription factors were scattered among 10 transcription factor modules (Figure 2D). Additionally, we mapped the expression signatures of the transcription factors from each module onto the corresponding cell types. The circos plots illustrated that in vertebrate ovaries, modules 1 and 4 were dedicated to the regulation of immune cells, while module 7 was exclusive to the governance of granulosa cells. Within mammalian ovaries, module 6 mediated the regulation of endothelial cells, module 9 influenced smooth muscle cells, and module 10 distinctly regulated granulosa cells (Figure 2E). Notably, differences were observed in the number of transcription factors within transcription factor modules across species, as well as in the connectivity between transcription factor modules and cell types. These findings indicate that ovarian cells from different species possess distinct transcriptional regulatory networks, likely reflecting evolutionary adaptations to species-specific biological processes. Utilizing the KEGG analysis of target genes within these conserved modules revealed enrichment in “Fc gamma R-mediated phagocytosis”, “Lysosome”, and “Phagosome” for modules 1 and 4, possibly implicating macrophage phagocytosis. Module 6 showed marked enrichment for the “Hippo signaling pathway”. In module 7, transcription factors influenced vertebrate granulosa cell functions via the “Thyroid hormone signaling pathway” and the “TGF-beta signaling pathway”. Module 9’s transcription factors uniquely modulated the “FoxO signaling pathway” and the “MAPK signaling pathway”. Lastly, the transcription factors in module 10 predominantly impacted mammalian granulosa cell functions through “Protein processing in the endoplasmic reticulum” (Figure 2F). Collectively, these findings corroborate the presence of conserved transcription factor modules in the vertebrate ovary, contributing to the regulation of biological processes in particular cell types.

### 3.4. Identification of Conserved and Characteristic Transcription Factors in Ovarian Endothelial Cells

**Figure 3:**
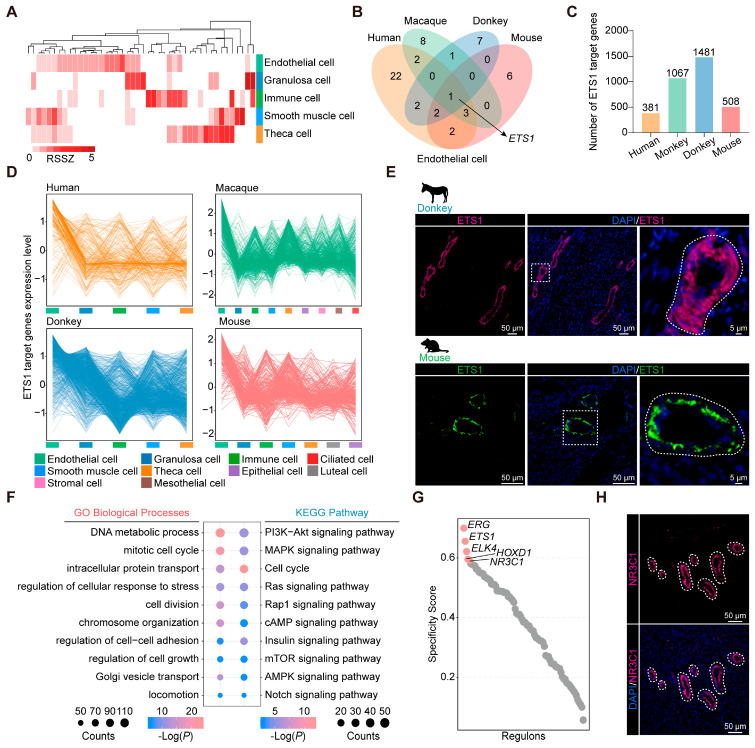
Identification of transcription factors in mammalian ovarian endothelial cells. (**A**) Heatmap showing the transcription factor RSSZ in different cell types of donkey ovary. (**B**) Venn diagram representing the overlap of orthologous transcription factors in four mammalian ovarian endothelial cells. (**C**) Histogram showing the number of ETS1 target genes in four mammalian ovaries. (**D**) Line chart showing the expression pattern of target genes of ETS1 in four mammalian ovaries. (**E**) Representative images of ETS1 staining of ovarian sections from donkeys and mice. Nucleus was counterstained with DAPI (blue). (**F**) GO and KEGG analysis of target genes of specific transcription factors in donkey ovarian endothelial cells. (**G**) Rank for regulons in donkey ovary endothelial cells based on regulon specificity score (RSS). (**H**) Representative images of NR3C1 staining of ovarian sections from donkeys. Nucleus was counterstained with DAPI (blue).

Follicle maturation coincides with the process of vascular remodeling in the ovary [6]. Additionally, considering the high transcriptional similarity among ovarian endothelial cells, we set out to pinpoint the conserved transcription factors underlying endothelial cell identity and functional characteristics. Based on the RSSZ analysis, we identified cell class-specific transcription factors, including 44 in donkeys, 131 in humans, 134 in macaques, 84 in mice, and 34 in zebrafish (Figure 3A and Figure S6).

We conducted a comparative analysis of characteristic transcription factors identified in endothelial cells derived from human, macaque, donkey, and mouse sources. Notably, our findings revealed a distinct overlap exclusively among the investigated species, particularly evident for ETS1 (Figure 3B). Subsequently, the target genes of *ETS1* with an importance measure (IM) exceeding 1 were extracted: 381 in humans, 1067 in macaques, 1481 in donkeys, and 508 in mice (Figure 3C). It is worth mentioning that most of the target genes of *ETS1* in mammalian ovaries are highly expressed in endothelial cells and are involved in biological functions related to endothelial cell development (Figure 3D and Figure S7A–D). Furthermore, immunostaining revealed ETS1 as a significant endothelial cell marker in both donkey and mouse ovaries (Figure 3E). Next, we performed GO and KEGG enrichment analysis on the target genes of specific transcription factors in donkey ovarian endothelial cells. We found these transcription factors to play a pivotal role in the regulation of endothelial cell growth, proliferation, and adhesion, these being processes fundamental to ovarian vascular remodeling [41] (Figure 3F). Using core gene regulatory network analysis, we discerned *ERG*, *ETS1*, *ELK4*, *HOXD1*, and *NR3C1* as the transcription factors exhibiting the highest specificity in association with donkey ovarian endothelial cells (Figure 3G). Notably, *ERG*, *ETS1*, *ELK4*, and *HOXD1* were identified as endothelial cell-specific transcription factors conserved across donkey, human, macaque, and mouse ovarian endothelial cells. In contrast, *NR3C1* was uniquely specific to donkey ovarian endothelial cells. The tissue immunofluorescence analysis further confirmed NR3C1 as a distinctive transcription factor for donkey ovarian endothelial cells, underscoring its potential as an important marker for this cell type (Figure 3H and Figure S7E).

### 3.5. Conserved Marker and Functional Genes in Granulosa Cells

**Figure 4:**
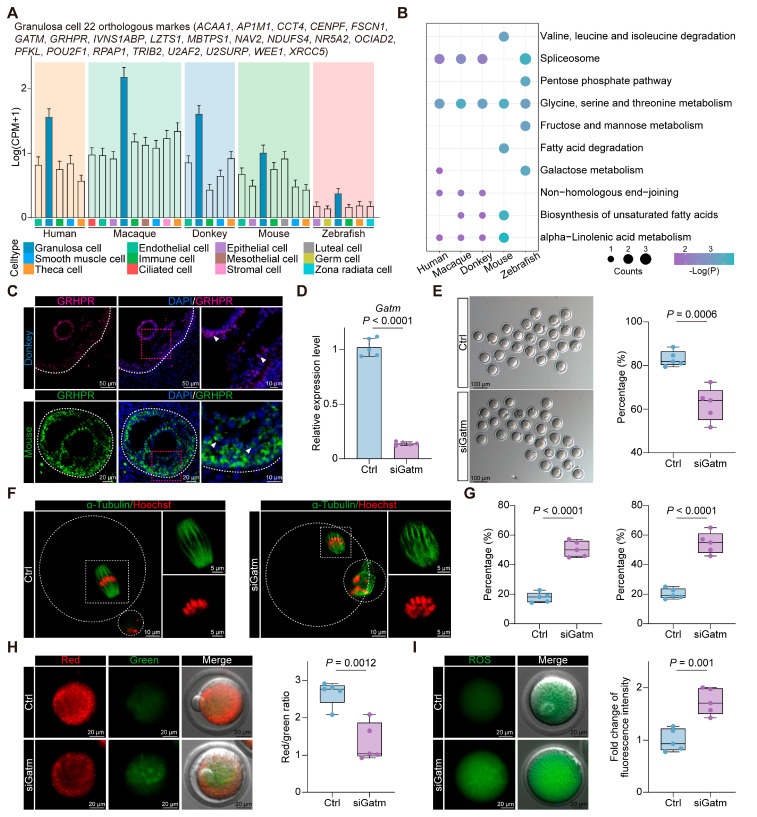
Molecular characterization of vertebrate granulosa cells. (**A**) Box plot showing the average expression levels of 22 orthologous marker genes in different cell types. (**B**) KEGG enrichment analysis of 22 orthologous marker genes from 5 species. (**C**) Representative images of GRHPR staining of ovarian sections from donkey and mouse. Nucleus was counterstained with DAPI (blue). White arrows indicate typical positively stained cells. (**D**) Gatm RNA levels in the Ctrl and siGatm groups. (**E**) Representative images of mature oocytes from both groups (**left**). The percentage of first PBE (**right**). Each group was repeated 5 times with each replicate containing 20–30 oocytes. (**F**) Representative pictures of normal and abnormal spindle morphologies and chromosome alignment. (**G**) Percentage of aberrant spindles (**left**) and misaligned chromosomes (**right**) in Ctrl and siGatm groups. Each group was repeated 5 times with each replicate containing 20–30 oocytes. (**H**) Mitochondrial membrane potential (ΔΨm) was detected by JC-1 staining in the Ctrl and siGatm groups (red, high ΔΨm; green, low ΔΨm) (**left**). The ratio of red to green fluorescence intensity was calculated in the Ctrl and siGatm groups (**right**). Each group was repeated 5 times with each replicate containing 20–30 oocytes. (**I**) Representative images of ROS levels detected by DCFH staining in the Ctrl and siGatm groups (**left**). The fluorescence intensity of ROS signals was measured in the Ctrl and siGatm groups (**right**). Each group was repeated 5 times with each replicate containing 20–30 oocytes.

To elucidate the conserved marker genes of granulosa cells across the vertebrates, we conducted intra-species difference analysis of ovarian cells within humans, macaques, donkeys, mice, and zebrafish, thereby isolating orthologous genes with specific expression in vertebrate granulosa cells (Figure 4A). Significantly, the identified genes were enriched in signaling pathways governing amino acid, lipid, and energy metabolism, with “glycine, serine, and threonine metabolism” notably pronounced in vertebrate granulosa cells, suggesting a conserved biological role (Figure 4B). Moreover, immunostaining with ovaries from both mice and donkeys substantiated the specific expression of GRHPR (a pivotal enzyme involved in “glycine, serine, and threonine metabolism”) within granulosa cells at the protein level (Figure 4C). We also identified another key enzyme in “glycine, serine, and threonine metabolism”, *GATM*. Studies have shown that *GATM* is highly expressed in the cumulus cells of COCs with a high capacity for blastocyst development and is considered a biomarker for high-potential COCs in cows [42]. To further confirm the role of *GATM* in oocyte maturation, we performed RNA interference on *Gatm* during the in vitro culture of mouse COCs. After 24 h, we measured the mRNA expression levels in cumulus cells, confirming the successful knockdown of *Gatm* (Figure 4D). Next, we quantified the first polar body extrusion (PBE) rate and observed that the decreased expression level of *Gatm* in cumulus cells negatively affected oocyte maturation (Figure 4E). Given that meiotic arrest is closely associated with spindle/chromosome structural defects, we evaluated the incidence of abnormal spindle/chromosome structures in two groups of oocytes. As expected, the incidence of spindle abnormalities and chromosome misalignment was significantly higher in the siGatm group (Figure 4F,G). These results indicate that *GATM* is essential for maintaining proper spindle/chromosome structure and oocyte maturation in vertebrates. Normal mitochondrial function is essential for oocyte maturation [43]. We assessed the mitochondrial membrane potential of oocytes in both groups and found that the decreased expression of *Gatm* in cumulus cells led to mitochondrial dysfunction in oocytes, as shown in Figure 4H. This mitochondrial dysfunction also resulted in increased oxidative stress levels in oocytes (Figure 4I). Therefore, we propose that *GATM* may play a crucial role in the regulation of mitochondrial function in vertebrate oocytes.

### 3.6. Comparative Analysis of Granulosa Cells from Different Species

**Figure 5:**
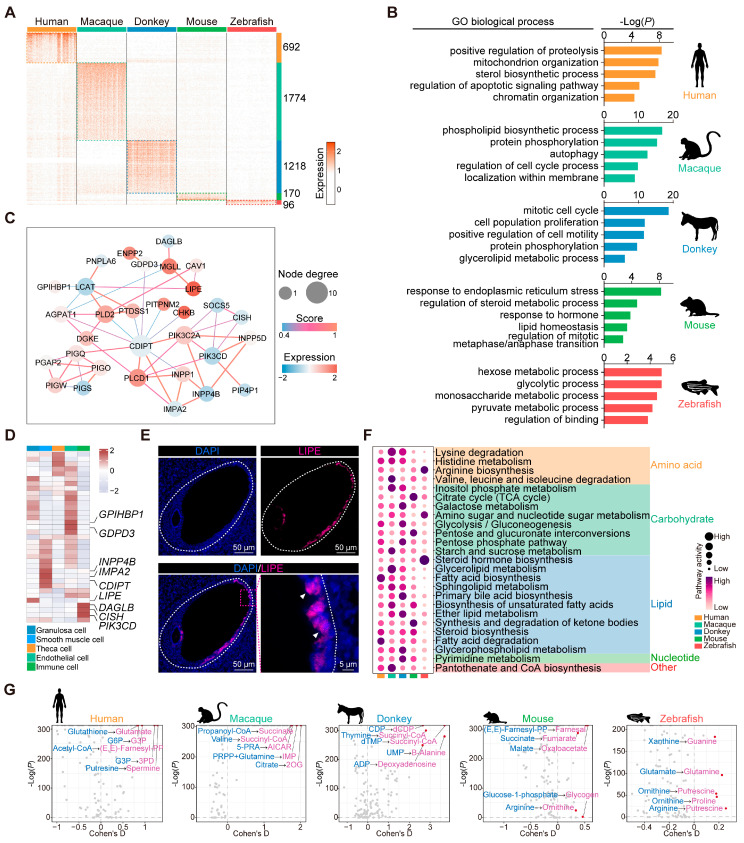
Characterization of donkey ovarian granulosa cells. (**A**) Heatmap of species-specific highly expressed genes in granulosa cells of five species. (**B**) GO enrichment analysis results of species-specific highly expressed genes in granulosa cells of five species. (**C**) Protein–protein interaction network diagram constructed based on key genes in the glycerolipid metabolic process. (**D**) Heatmap showing the average expression levels of key genes in the glycerolipid metabolic process of various types of cells in donkey ovary. (**E**) Representative images of LIPE staining of ovarian sections from donkey. Nucleus was counterstained with DAPI (blue). White arrows indicate typical positively stained cells. (**F**) Bubble plot showing the score of metabolic pathway activity in granulosa cells of different species. (**G**) Volcano plot showing differences in metabolic fluxes of granulosa cells in different species.

Our subsequent analysis delved into the transcriptomic disparities of granulosa cells among the species, revealing species-specific genes: 692 in humans, 1774 in macaques, 1218 in donkeys, 170 in mice, and 96 in zebrafish (Figure 5A). The GO enrichment analysis conducted on these species-specific homologous genes unveiled that signaling pathways pertaining to energy metabolism are predominantly enriched in zebrafish, whereas in mammals, they are primarily associated with cell proliferation and lipid metabolism. This observation suggests the existence of distinct functional disparities in the granulosa cells of zebrafish compared with mammalian species (Figure 5B). Additionally, our attention was directed towards the enrichment of the term “glycerolipid metabolic process” specifically observed in donkey granulosa cells, suggesting a potentially distinctive biological function within this cellular context. By mapping the genes associated with this process onto a protein–protein interaction network, we noted that Lipase E (*LIPE*), *PLCD1*, *MGLL*, *PLD2*, and *DGKE* were central to the network (Figure 5C). Notably, we presented the average expression levels of these genes across the various types of donkey ovarian cells in the form of heat maps. Several genes exhibited cell type-specific high expression patterns. Specifically, *GPIHBP1* and *GDPD3* demonstrated elevated expression levels in endothelial cells, while *INPP4B*, *IMPA2*, and *CDIPT* displayed heightened expression in smooth muscle cells. Additionally, *DAGLB*, *CISH*, and *PIK3CD* exhibited relatively higher expression levels in immune cells, whereas *LIPE* demonstrated specificity in granulosa cells (Figure 5D). Furthermore, our immunofluorescence results from donkey ovary tissue confirmed the granulosa cell-specific expression characteristics of LIPE (Figure 5E). *LIPE*, also known as hormone-sensitive lipase (*HSL*), plays a critical role in steroidogenic tissues by interacting with lipid droplets and catalyzing the hydrolysis of cholesterol esters. This process generates essential cholesterol substrates required for the rapid synthesis of steroid hormones [44,45]. Its expression and functional significance in donkey granulosa cells suggest that *LIPE* serves as a specific marker, highlighting its pivotal role in granulosa cell steroidogenesis.

Granulosa cells play a pivotal role in establishing the metabolic environment necessary for oocyte maturation [46]. To elucidate the evolutionary adaptations of granulosa cell metabolism, we analyzed metabolic pathway activity in granulosa cells from various species using a metabolic landscape pipeline [28]. In humans, the granulosa cells exhibited significantly higher fatty acid metabolism activity, while granulosa cells from macaques showed heightened activities in pathways such as inositol phosphate metabolism and pantothenate and CoA biosynthesis. In the donkey granulosa cells, bile acid metabolism, glycerophospholipid metabolism, and pyrimidine metabolism were notably more active. The mouse granulosa cells demonstrated significantly elevated carbohydrate metabolism, including the TCA cycle. In contrast, the zebrafish granulosa cells displayed higher activity in steroid hormone and arginine metabolism pathways (Figure 5F). Significant differences were also observed in the metabolic flux of granulosa cells across the species. The human granulosa cells exhibited a higher utilization of metabolites such as glutathione and acetyl-CoA. In macaques, the granulosa cells demonstrated elevated citrate-to-2OG metabolic reactions. The donkey granulosa cells showed pronounced metabolic activity within the pyrimidine metabolism pathway. The mouse granulosa cells displayed distinct differences in metabolites associated with the TCA cycle, while the zebrafish granulosa cells exhibited stronger metabolic activity involving putrescine (Figure 5G). In summary, our findings highlight that granulosa cells across different species exhibit distinct metabolic profiles, reflecting adaptations to their respective reproductive requirements.

### 3.7. Conservation and Characteristics of Theca Cell Development in Diverse Species

**Figure 6:**
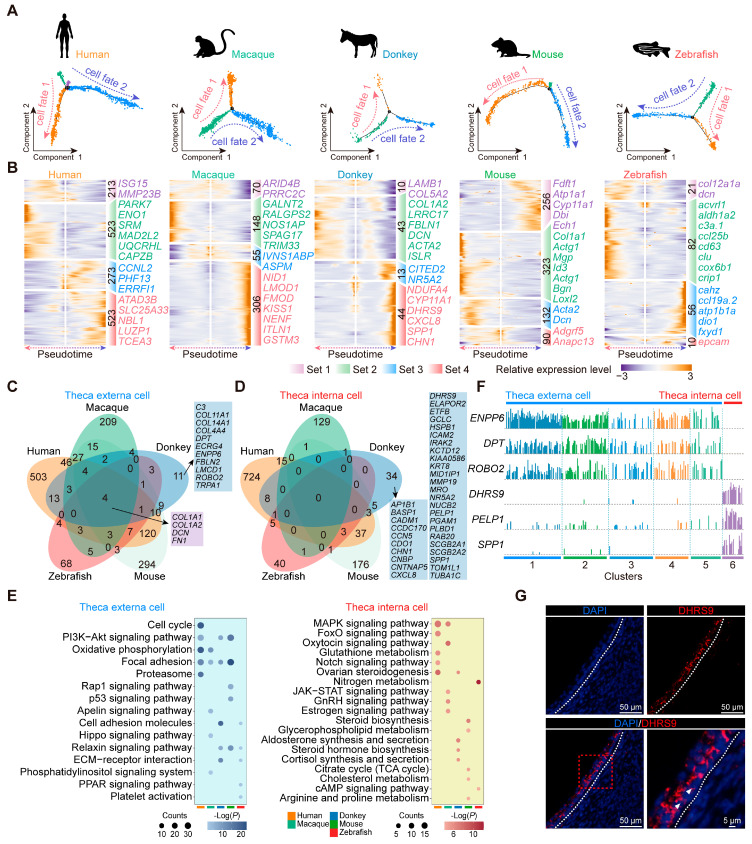
Developmental characteristics of donkey theca cells. (**A**) Pseudotime trajectories of theca cells in humans, macaques, donkeys, mice, and zebrafish. (**B**) Heatmap representing gene expression dynamics during pseudotime ordering of theca cells. (**C**) Venn diagram representing the overlap of orthologous genes in theca externa cells of five vertebrate species. (**D**) Venn diagram representing the overlap of orthologous genes in theca interna cells of five vertebrate species. (**E**) The KEGG enrichment analysis outcomes of pivotal genes governing the development of both theca externa and theca interna cells within follicles across five species. (**F**) Expression levels of *DHRS9*, *DPT*, *ENPP6*, *PELP1*, *ROBO2,* and SPP1 in donkey ovarian theca cells. (**G**) Representative images of DHRS9 staining of ovarian sections from donkey. Nucleus were counterstained with DAPI (blue). White arrows indicate typical positively stained cells.

Theca cells consist of theca interna cells and theca externa cells, each with distinct functional roles [47]. We analyzed the evolutionary conservation and unique developmental traits of theca cells by reconstructing their pseudotime trajectories across different species (Figure 6A). The pseudotime trajectories displayed a consistent pattern across all five species, depicting the differentiation of both cell fates. This consistency suggests that in adult vertebrates, theca interna cells and theca externa cells may both originate from a common progenitor [47]. Subsequently, we executed a differential analysis predicated on the pseudotime ordering of theca cells, pinpointing four distinct gene sets that exhibited heightened expression at various developmental stages, and displayed the most significantly different marker genes at each stage (Figure 6B). Based on the GO enrichment analysis of gene sets across the five species, we observed distinct patterns in gene expression associated with two cell fates. The gene sets exhibiting sustained elevation in expression levels within cell fate 1 were enriched for biological functions related to metabolism, specifically lipid metabolism and energy metabolism. Then, the gene sets characterized by continuous elevation in expression levels within cell fate 2 were enriched for biological processes encompassing matrix organization, cell adhesion, and mitosis (Figure S8). Furthermore, we noted that *STAR* [21] (a marker gene for theca interna cells) exhibited significant expression in the cell fate 1 branch, while *COL1A1* [48] (a marker gene for fibroblasts) was highly expressed in the cell fate 2 branch across the vertebrates (Figure S9). Because the theca interna contains theca endocrine cells and the externa is a fibrous, connective tissue layer derived from fibroblast-like cells [47], we believe that in vertebrates, cell fate 1 represents the developmental trajectory of theca interna cells, whereas cell fate 2 corresponds to the developmental trajectory of theca externa cells.

To delve deeper into the distinctive developmental characteristics of donkey theca cells relative to those of the other vertebrates, we translated the key genes implicated in the development of both cell types into their human orthologs. Through comparative analysis, we identified *COL1A1*, *COL1A2*, *DCN*, and *FN1* as the key conserved genes involved in the development of vertebrate theca externa cells. In contrast, genes such as *C3*, *COL11A1*, *DPT*, and *ENPP6* exhibited species-specific expression patterns, uniquely associated with the development of donkey theca externa cells. (Figure 6C). Notably, our study did not identify any universally conserved genes associated with theca interna cell development across the vertebrates. This observation suggests that theca interna cells may have undergone strong evolutionary selection, leading to significant divergence in the genomic composition related to their developmental functions among different species (Figure 6D). Additionally, within this context, we identified several genes uniquely associated with theca interna cell development in donkeys, including *CDO1*, *DHRS9*, *ELAPOR2*, and *ETFB*, among others (Figure 6D). Our study also concentrated on signal transduction pathways in theca cells, observing a considerable enrichment of “focal adhesion” in mammalian theca externa cells—a reflection of its conserved and crucial role in their development. Notably, “aldosterone synthesis and secretion” exhibited specific enrichment in donkey theca interna cells (Figure 6E). Ultimately, the cluster analysis and gene expression signature analysis were conducted on donkey theca cells to identify specific markers exhibiting high expression in donkey theca externa and theca interna cells. Specifically, *ENPP6*, *DPT*, and *ROBO2* exhibited significantly high expression levels in theca externa cells, while *DHRS9*, *PELP1*, and *SPP1* showed specific expression in donkey theca interna cells (Figure 6F). The immunofluorescence staining further validated the specific expression of DHRS9 within theca interna cells in donkeys (Figure 6G). Taken together, these findings suggest that donkey theca cells harbor species-specific key regulatory genes.

In addition, the species-specific genes in theca externa cells and theca interna cells of the five vertebrates were identified through differential analysis, leading to the construction of a transcriptional regulatory network based on these species-specific gene sets (Figure S10). This analysis revealed the key regulators of theca cells unique to each species. Specifically, *HNRNPA1*, *H2AFZ*, *SRP9*, *ARID5B*, and *CYCS* were identified as key regulators in human theca externa cells; *NR5A2*, *PEG3*, *BDP1*, *TBL1XR1*, and *PPARG* in macaque theca externa cells; *EEF1D*, *TCF21*, *ANXA1*, *CANX*, and *ANXA4* in donkey theca externa cells; *NME1*, *MYLK*, *AKR1A1*, *EGR1*, and *UQCRB* in mouse theca externa cells; and *PHLDA2*, *LTF*, *CEBPB*, *ENO1*, and *EIF5A2* in zebrafish theca externa cells. In theca interna cells, *HMGB1*, *YBX1*, *HES1*, *HSPA5*, and *FHL2* were identified as key regulators in humans; *RUNX2*, *NUAK1*, *ESR2*, *MKX*, and *PBX1* in macaques; *RAN*, *ZNF140*, *MTHFD1*, *KDM5A*, and *MAF* in donkeys; *NR5A1*, *SUCLG1*, *PSMA6*, *SMPX*, and *TSN* in mice; and *FOS*, *ID2*, *JDP2*, *CPEB1*, and *HMGB3* in zebrafish. Taken together, these findings underscore the unique transcriptional signatures of theca cells across the vertebrate species.

### 3.8. Interaction Networks of Ovarian Cells in Each Species

**Figure 7:**
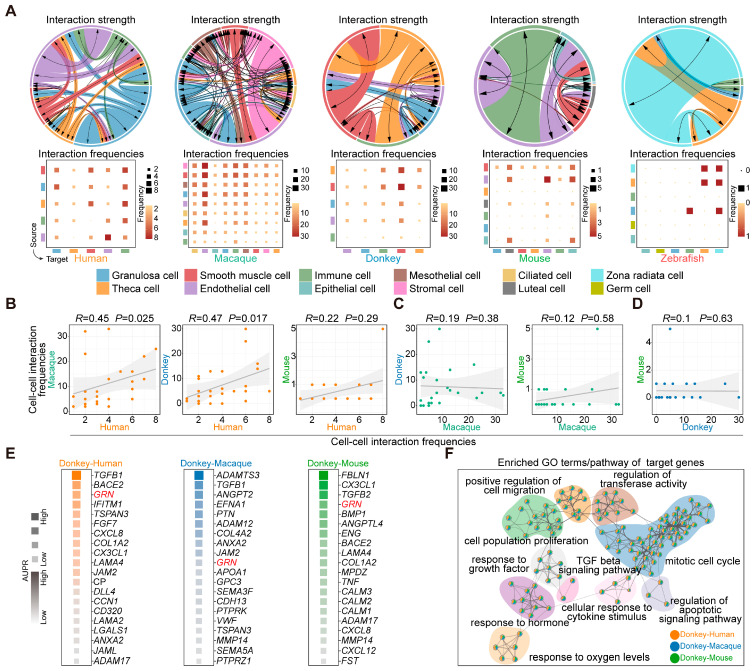
Interactions between ovarian cells across species. (**A**) Circos plots (upper) and heatmaps (below) illustrate the strength and frequency of interactions among major cell types within the ovaries of humans, macaques, donkeys, mice, and zebrafish. (**B**) Scatter plots show Spearman’s rank correlation of cell–cell interaction frequencies between human and macaque, human and donkey, and human and mouse. (**C**) Scatter plots show Spearman’s rank correlation of cell–cell interaction frequencies between macaque and donkey, and macaque and mouse. (**D**) Scatter plots show Spearman’s rank correlation of cell–cell interaction frequencies between donkey and mouse. *p*-values are provided (two-sided Spearman’s correlation test). The fitted line and standard errors with 95% confidence intervals are shown. (**E**) Plots derived from NicheNet analysis showing predicted ligand activity for top ligands (ordered by Pearson’s correlation coefficient) in donkey and human, donkey and macaque, and donkey and mouse. (**F**) Network plot showing enriched GO terms/pathways for ligand-target genes.

To decipher the dynamics of ovarian cell communication, we used CellChat to calculate the strength and frequency of the interactions between various cell types [32]. Based on these results, we observed strong intercellular interactions between smooth muscle cells and other cell types across the mammalian ovaries. Moreover, the human ovary exhibits strong interactions between theca cells and immune cells, while the macaque ovary demonstrates strong interactions between endothelial cells and other cell types. Notably, the donkey ovary exhibits a pattern akin to that of the human ovary, characterized by robust interactions between theca cells and immune cells. Additionally, in the mouse ovary, there were notable interactions between epithelial cells and other cell types (Figure 7A). Subsequently, we conducted a correlation analysis on the frequency of cell–cell interactions across different species. The findings revealed that the patterns of cell-to-cell interactions in human and macaque, and human and donkey ovaries exhibited greater similarity compared with those observed in mice (Figure 7B–D).

Considering the pivotal role of granulosa cells in oocyte development, we conducted NicheNet analysis to comprehensively predict the ligand-target gene network between ovarian cells (endothelial cells, immune cells, smooth muscle cells, and theca cells) and granulosa cells. Through comparative analysis of the ligand-target gene matrices between donkey and human, donkey and macaque, as well as donkey and mouse, we identified the top 20 ligands exhibiting species-specific high activity in donkey ovarian cells across the three groups (Figure 7E). Simultaneously, we extracted the target genes regulated by ligands within granulosa cells for subsequent enrichment analysis. According to the findings from the enrichment analysis, we noted that the species-specific ligands identified in donkey ovarian cells specifically targeted and regulated genes associated with cell proliferation and migration in granulosa cells (Figure 7F). Notably, we observed elevated activity of Granulin (*GRN*) across all three groups. *GRN* is a multifunctional protein that functions as a key regulator of lysosomal activity and acts as a growth factor critically involved in cell proliferation [49,50]. This finding underscores its potential importance as a species-specific factor regulating granulosa cell fate determination in donkeys.

## 4. Discussion

The increasing demand for donkeys in China’s donkey hide gelatin trade and donkey meat market, coupled with the burgeoning donkey dairy industry in Eastern Europe, underscores the need to improve the reproductive rate of this species [51]. Currently, the development of the donkey industry faces significant challenges due to biological constraints, including a prolonged reproductive cycle, slow reproduction rate, low conception rate, high abortion rate, and reduced survival rate [52,53]. Notably, comparative studies have highlighted substantial differences in reproductive performance between donkeys and horses, which are both large, single-birth animals. For instance, research by Bocci et al. reported a markedly higher conception rate in horses (67%) compared with donkeys (37%) under identical environmental conditions [54]. This indicates that there is still the potential to enhance the reproductive capacity of the donkey. As ovarian function significantly influences an animal’s reproductive capabilities, by encompassing factors such as estrus and fetal survival rate, a thorough analysis of the molecular characteristics of donkey ovarian cells is essential to unlock their reproductive potential. In this study, we initially constructed a molecular characterization map of the donkey ovary at the single-cell level. By integrating scRNA-seq data from zebrafish, mice, macaques, and humans, we elucidated significant regulatory factors that are conserved and characterized in donkey ovarian cells from an evolutionary standpoint.

MetaNeighbor analysis employs AUROC scores to quantify the similarity between pairs of cell types, making it a widely utilized tool in cross-species scRNA-seq analysis [25]. Using this approach, we identified that endothelial cells, immune cells, epithelial cells, and smooth muscle cells were highly conserved across vertebrate ovaries, consistent with findings by Li et al. in whole-body scRNA-seq data from humans, mice, and zebrafish [55]. This indicated a conserved expression program in these cell types within the vertebrate ovaries. We further investigated the homology among vertebrate ovarian cells from the perspective of transcription factor regulatory programs, and elucidated that transcription factor modules regulated cellular functions through specific signaling pathways. Among these, we found that the “thyroid hormone signaling pathway” served as a crucial pathway for the conserved transcription factor module regulation of granulosa cell function in vertebrates. It has been reported that thyroid hormone transport proteins and receptors are expressed in the ovary, and that thyroid hormones can directly act on granulosa cells [56]. In the study by Wang et al., the combination of triiodothyronine (T3) and FSH was found to regulate granulosa cell proliferation and inhibit granulosa cell apoptosis through the PI3K/AKT signaling pathway [57]. Furthermore, our analysis revealed that the transcription factor module regulates the expression of *FOXO1*, *PFKP*, and *RAF1* within the “thyroid hormone signaling pathway”. Specifically, *FOXO1* plays a pivotal role in modulating granulosa cell apoptosis, autophagy, oxidative stress, and hormone secretion [58,59,60]. *PFKP* has been identified as a key regulator of glycolysis in granulosa cells [61], while *RAF1* is involved in the synthesis and secretion of estradiol [62]. Remarkably, the enrichment of “protein processing in endoplasmic reticulum” was confined solely to the conserved transcription factor module within mammalian granulosa cells. This aligns with Miyuki’s proposition that endoplasmic reticulum stress serves as a pivotal regulator of the ovarian follicle microenvironment [63].

The cyclic vascular morphogenesis initiated by endothelial cells intricately intertwines with ovarian function [64]. Our study revealed *ETS1* as a conserved transcription factor present in mammalian ovarian endothelial cells. In mouse models, *ETS1* has been identified as essential for the maintenance of endothelial cell survival and the regulation of embryonic vasculogenesis [65]. Furthermore, the endothelial cell-specific ablation of *ETS1* has been shown to detrimentally impact the development of coronary vessels [66]. Moreover, insights from Toshiaki’s research indicate a correlation between cyclic variations in *ETS1* mRNA expression and the modulation of luteal function [67]. Consequently, our conjecture posits that the presence of *ETS1* is imperative for orchestrating endothelial cell development within the mammalian ovary, thereby facilitating the preservation of ovarian homeostasis. Our investigation also centered on the species-specific transcription factor *NR3C1* within endothelial cells of the donkey ovary. *NR3C1* encodes the glucocorticoid receptor (GR), and empirical data indicated that endogenous corticosterone mediates the mitigation of vascular inflammation through the activation of endothelial cell GR [68]. On the other hand, the knockout of *NR3C1* in endothelial cells was observed to elicit enhanced angiogenesis [69]. These findings provide evidence suggesting that *NR3C1* may take part in the modulation of the vascular milieu and the regulation of blood vessel development within donkey ovaries.

The metabolic synergy between granulosa cells and oocytes predominates during oocyte maturation [70]. In our investigation, “glycine, serine, and threonine metabolism” exhibited significant enrichment within vertebrate granulosa cells. The results indicated that the “glycine, serine, and threonine metabolism” pathway serves as a crucial metabolic hub. On the one hand, the reaction catalyzed by serine hydroxymethyltransferase (SHMT) generated folic acid through the folate cycle. Alternatively, serine plays a pivotal role in sulfur metabolism while also actively participating in the intricate processes of glycerophospholipid metabolism [71]. It is noteworthy that lysophosphatidic acid (LPA) and lysophosphatidylcholine (LPC) serve as pivotal metabolites within the glycerophospholipid metabolic pathway. LPA is known to facilitate the expansion of the COC and the maturation of oocytes, whereas LPC plays a crucial role in the formation of follicular sinuses and the maturation of oocytes [72,73]. In light of our findings, it can be speculated that “glycine, serine, and threonine metabolism” plays a pivotal role in the functional regulation of vertebrate granulosa cells. Equally important, this study unveiled, for the first time, the notable and specific expression of *LIPE* within donkey granulosa cells. *LIPE* can convert cholesterol esters into free cholesterol to support steroid hormone production, which is essential for granulosa cells to complete estrogen secretion and support follicle development [74]. In our examination of the ligand–receptor communication network within mammalian ovaries, we noted that *GRN*, serving as a ligand signal that targets and regulates granulosa cells, exhibited elevated activity levels in donkey ovaries compared with those of humans, macaques, and mice. In their 2023 study, Cui et al. discovered that *GRN* signaling functions to inhibit cell differentiation in planarians [75]. Notably, the granulosa cells residing within growing follicles are recognized as non-terminally differentiated cells. This observation implies a pivotal role for *GRN* in the regulation of granulosa cell differentiation [76]. Thus, we believe that targeting *LIPE* and *GRN* could offer a novel strategy to tap the reproductive potential of donkeys.

Theca cells play a multifaceted role, encompassing the preservation of follicular structural integrity, provisioning of nutrients to granulosa cells and oocytes, as well as the secretion of endocrine regulatory factors, including androgens, and growth regulatory factors exemplified by BMP and TGF-β [77]. Diverse functionalities emerge from the division of the theca into theca externa cells and theca interna cells, a structural organization observed ubiquitously in vertebrates. Moreover, our findings underscore the significance of “focal adhesion” in mammalian theca externa cells. We speculate that this phenomenon is intricately linked to the ability of “focal adhesion” to facilitate cellular adhesion to the extracellular matrix (ECM) and promote cellular motility [78]. It is also worth noting that *DHRS9* specifically regulates the function of donkey theca interna cells. Previous studies have highlighted the involvement of *DHRS9* in androgen and progesterone steroidogenesis [79]. Given that theca interna cells are pivotal for androgen production, the role of *DHRS9* in this context is particularly significant. Moreover, *DHRS9* has been implicated in retinol metabolism. Intriguingly, retinol has been shown to enhance androgen synthesis in theca interna cells [80]. These results point to *DHRS9* playing an important role in donkey theca interna cells and being species-specific.

The observed differences in gene expression patterns between species may be influenced by the variable stages of estrous cycle and follicular development at the time of sample collection. Although zebrafish lack a well-defined estrous cycle, mammals including humans, macaques, and mice exhibit cyclical reproductive patterns comprising four distinct phases, proestrus, estrus, metestrus, and diestrus, each characterized by unique hormonal profiles and follicular dynamics [81]. During proestrus and estrus, the developing follicles exhibit high estradiol production and active granulosa cell proliferation, while during metestrus and diestrus, corpus luteum formation and progesterone dominance occur [82]. These cyclical changes significantly impact ovarian gene expression profiles. Furthermore, the heterogeneity of follicles at different developmental stages (primordial, primary, secondary, and antral follicles) within each ovary contributes additional complexity to the transcriptomic landscape [83], because each follicular stage expresses distinct sets of genes related to oocyte maturation, granulosa cell differentiation, and theca cell function. Given these, the variability in estrous cycle synchronization and follicular stage distribution among our samples may partially explain the species-specific differences observed in our analysis. Future studies incorporating precise estrous cycle staging and follicular classification could reveal more homogeneous gene expression patterns, and potentially uncover conserved regulatory mechanisms with higher accuracy.

## 5. Conclusions

In this study, we comprehensively investigated the characteristic genes, transcription factor regulatory networks, signaling pathways, and ligand–receptor networks of adult donkey ovarian cells, utilizing diverse model organisms. Although this analysis offers valuable evolutionary insights, certain methodological limitations should be acknowledged. Due to ethical constraints and limited sample availability, the single-cell transcriptomic analysis did not encompass the entire ovarian tissue architecture for all species examined. Despite this limitation, our findings provide a systematic characterization of both conserved and species-specific adaptations in ovarian cell types, yielding important molecular insights into the mechanisms underlying interspecies differences in reproductive efficiency.

## Data Availability

The data generated in this study have been deposited in the Genome Sequence Archive (GSA, https://ngdc.cncb.ac.cn/gsa (accessed on 7 August 2024)) under the accession number CRA018235.

## Supplementary Materials

The following supporting information can be downloaded at: https://www.mdpi.com/article/10.3390/ani15121761/s1, Figure S1. Identification of donkey ovarian cell types by single-cell transcriptomics. Figure S2. Annotation of cell identity in zebrafish ovary. Figure S3. Identification of mouse ovarian cells. Figure S4. Annotation of cell identity in macaque ovary. Figure S5. Identification of human ovarian cells. Figure S6. Identification of cell type-specific transcription factors in vertebrate ovary. Figure S7. Function and localization of transcription factors in mammalian ovarian endothelial cells. Figure S8. Molecular characterization of theca cell subsets. Figure S9. Pseudotime developmental trajectories of vertebrate theca cells. Figure S10. Characteristics of theca cells of different species. Table S1. Antibodies used in this paper. Table S2. Sequences of siRNA oligonucleotides. Table S3. Primers used for RT-qPCR.

## Abbreviations

The following abbreviations are used in this manuscript.

AUROC	Area under the receiver operator characteristic curve
BSA	Bovine serum albumin
COC	Cumulus–oocyte complex
CSI	Connection similarity index
ECM	Extracellular matrix
GO	Gene Ontology
GR	Glucocorticoid receptor
KEGG	Kyoto Encyclopedia of Genes and Genomes
LPA	Lysophosphatidic acid
NC	Negative control
PCC	Pearson’s correlation coefficient
PFA	Paraformaldehyde
PMSG	Pregnant mare serum gonadotropin
ROS	Reactive oxygen species
RPCA	Reciprocal principal component analysis
RSSZ	Z-score normalized regulon specificity score
RT-qPCR	Real time quantitative PCR
scRNA-seq	Single-cell RNA sequencing
SDS–PAGE	SDS–polyacrylamide gel electrophoresis
SHMT	Serine hydroxymethyltransferase
siRNA	Small interfering RNA
T3	Triiodothyronine
