# Supplementary material for: The Transcriptomic Signature of Donkey Ovarian Tissue Revealed by Cross-Species Comparative Analysis at Single-Cell Resolution
Source: Animals (Basel). 2025 Jun 14;15(12):1761. doi: 10.3390/ani15121761 (PMC12189512; doi:10.3390/ani15121761)
Supplement: Supplementary file 1 [file animals-15-01761-s001.zip › animals-3636123-supplementary.pdf]

animals

MDPI

Supplementary Materials

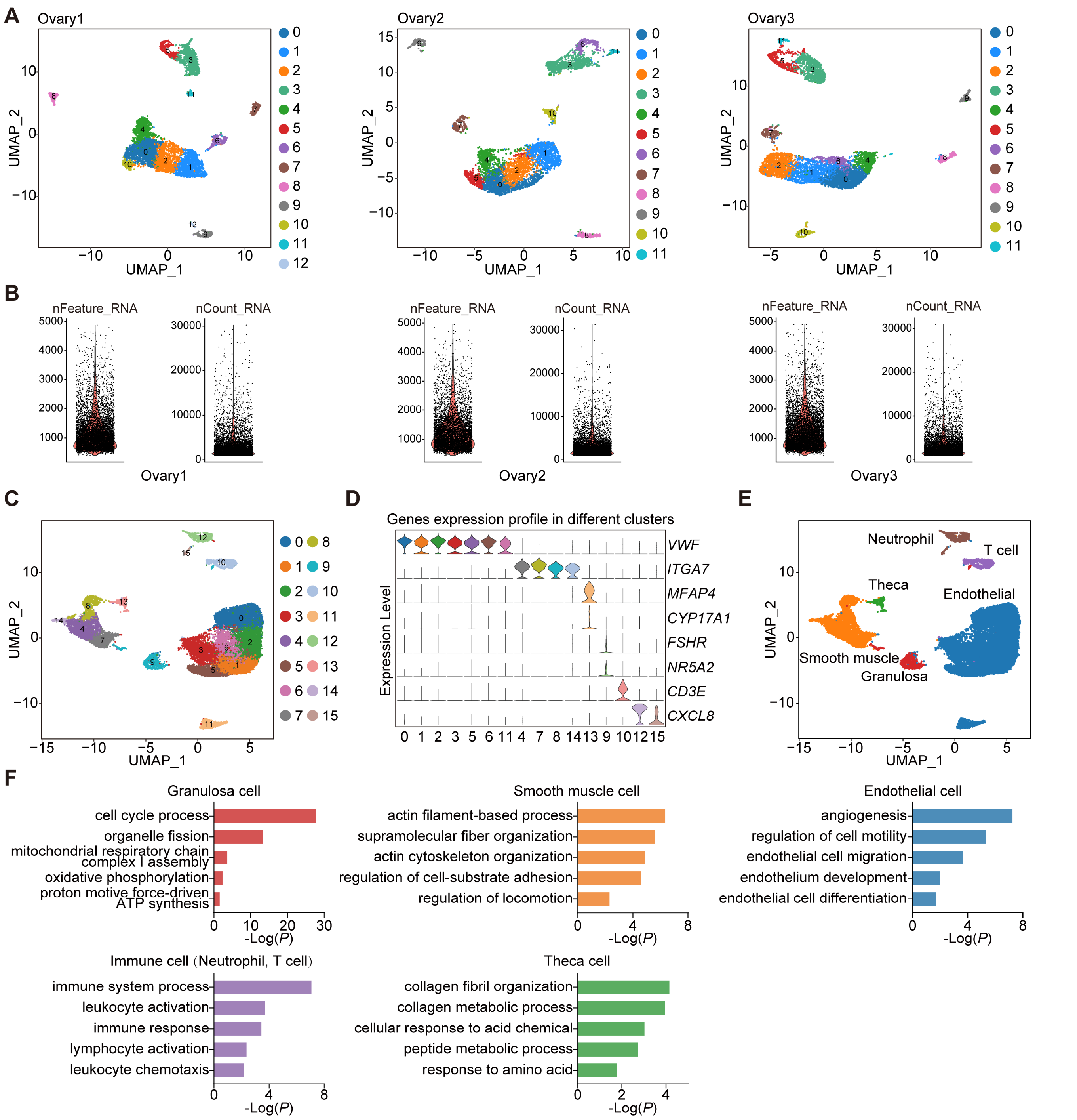

Supplementary Figure S1. Identification of donkey ovarian cell types by single-cell transcriptomics (A) UMAP plot of ovarian cells based on sample group. (B) Quality control of single-cell transcriptome data. (C) UMAP diagram after integrating different samples. (D) Representative gene expression levels for each cell type. (E) UMAP plot was used to visualize six ovarian cell types. (F) Representative GO terms for different cell type specific highly expressed gene sets.

 https://doi.org/10.3390/ani15121761

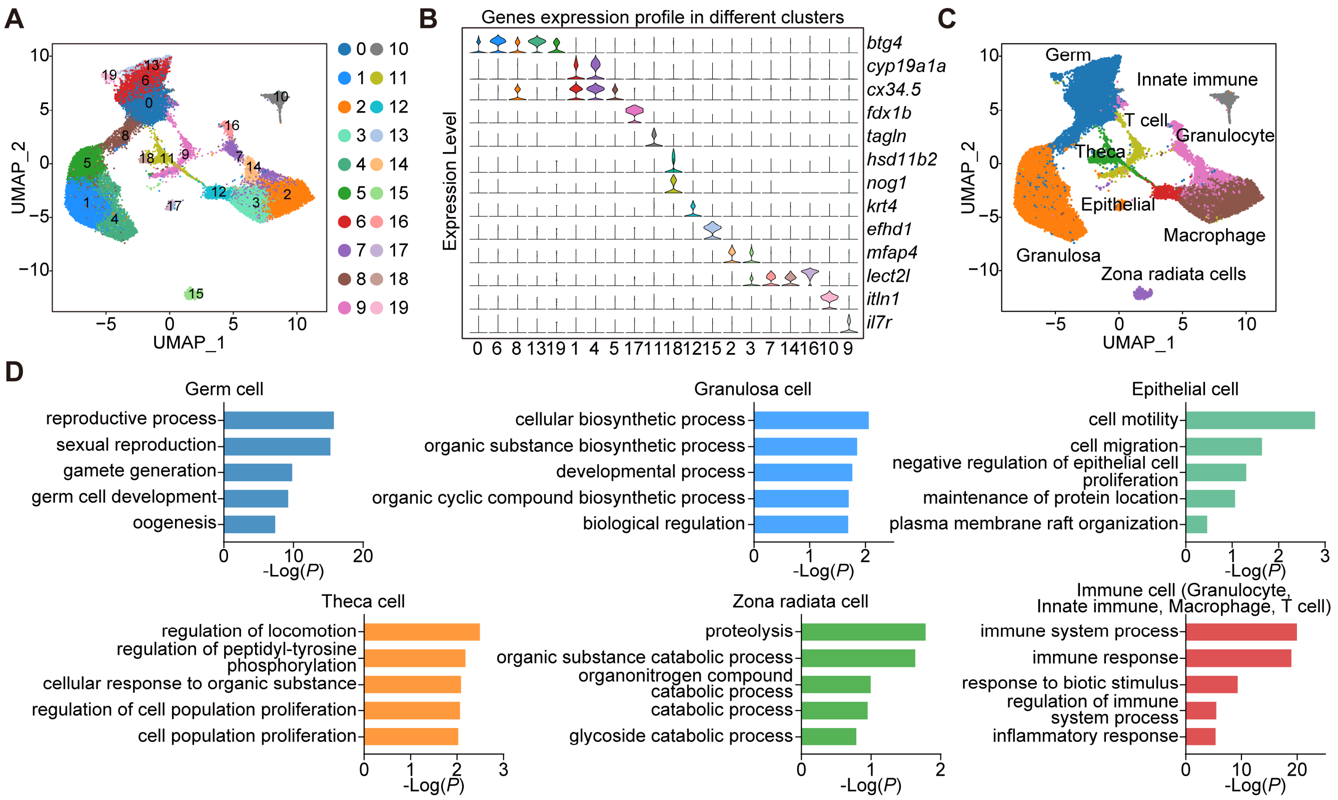

Supplementary Figure S2. Annotation of cell identity in zebrafish ovary (A) The UMAP map revealed 20 clusters representing major ovarian cell types. (B) Violin plots of the expression level of cell marker genes in each type. (C) UMAP diagram showed the distribution characteristics of nine types of ovarian cells. (D) Representative GO terms for different cell type specific sets of highly expressed genes.

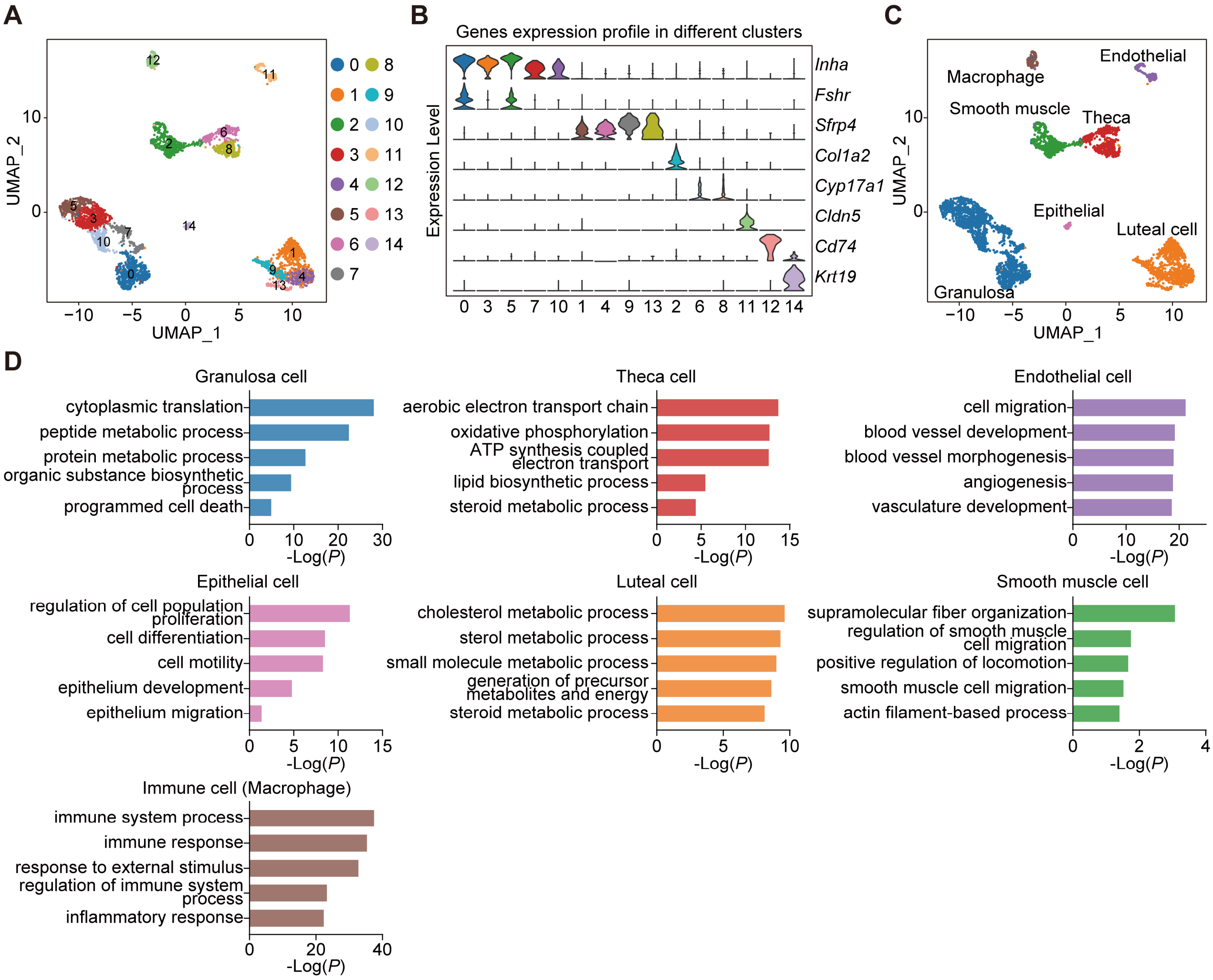

Supplementary Figure S3. Annotation of cell identity in mouse ovary (A) The UMAP map revealed 15 clusters representing major ovarian cell types. (B) Violin plots of the expression level of cell marker genes in each type. (C) UMAP diagram showed the distribution characteristics of seven types of ovarian cells. (D) Representative GO terms for different cell type specific sets of highly expressed genes.

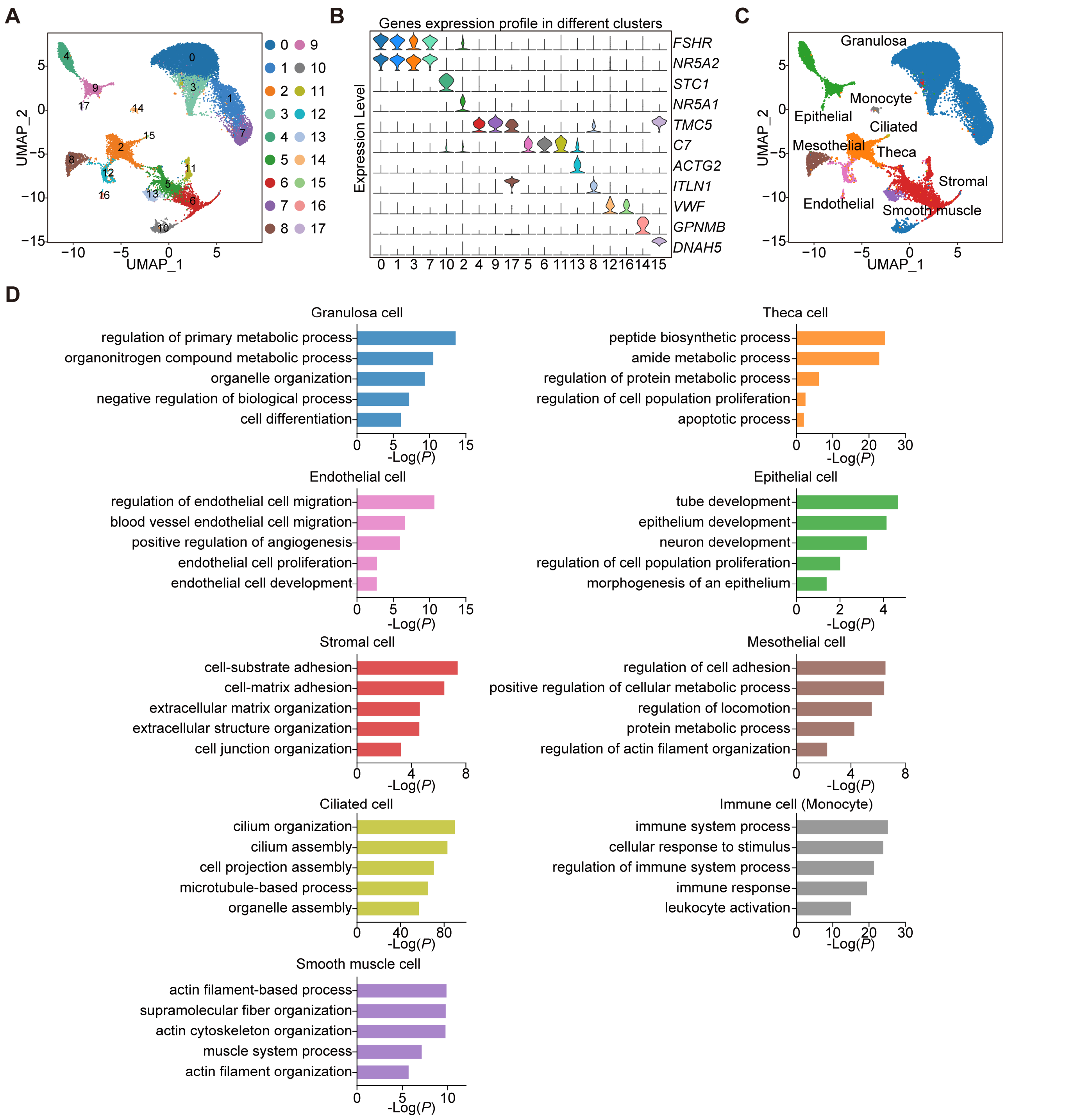

Supplementary Figure S4. Annotation of cell identity in macaque ovary (A) UMAP scatterplot visualizing cell clusters. (B) Violin plots showing expression of one representative differential expressed gene for each cluster. (C) Clustering results of 9 types of ovarian cells. (D) Representative GO terms for different cell type specific highly expressed gene sets.

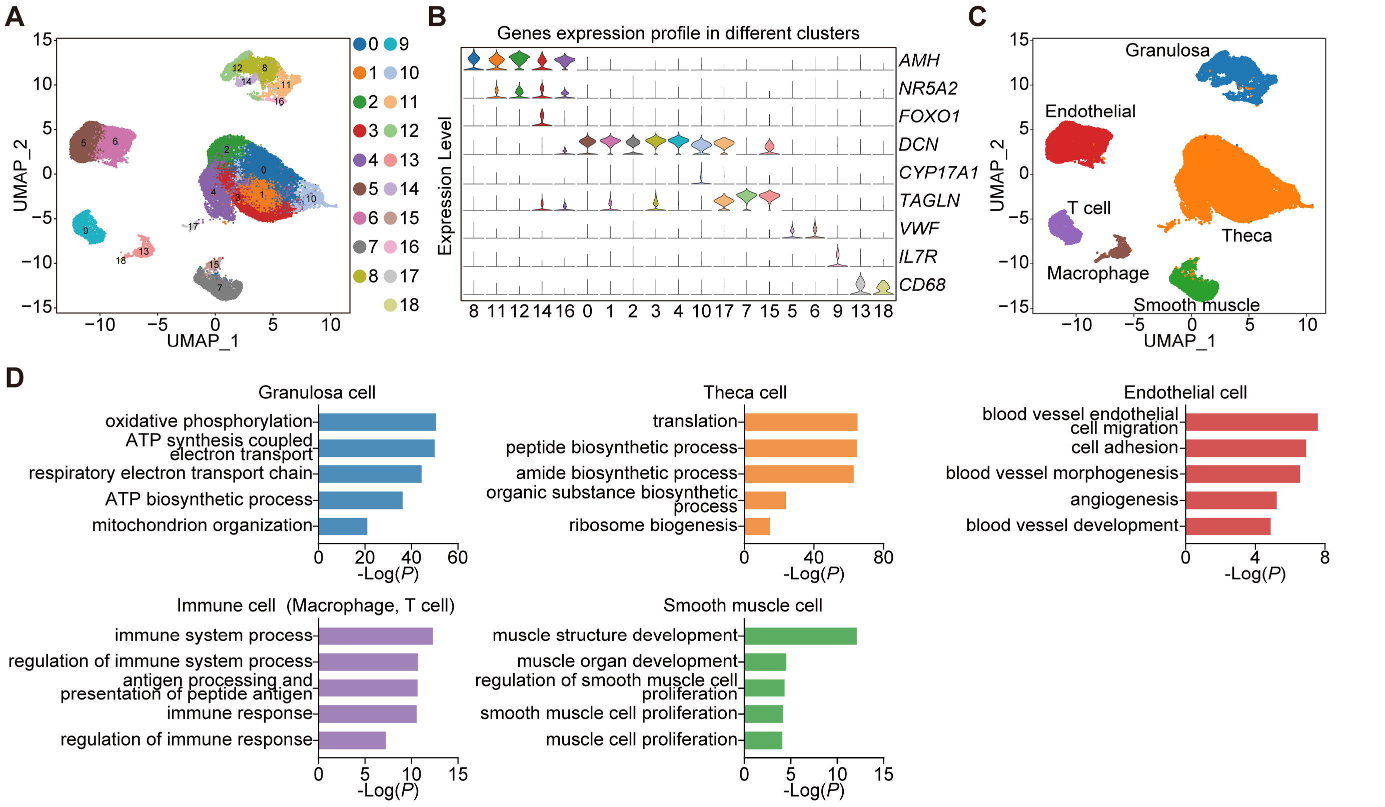

Supplementary Figure S5. Identification of human ovarian cells (A) The UMAP map revealed 19 clusters representing major ovarian cell types. (B) Violin plots showing expression of one representative differential expressed gene for each cluster. (C) UMAP diagram showed the distribution characteristics of six types of ovarian cells. (D) Representative GO terms for different cell type specific highly expressed gene sets.

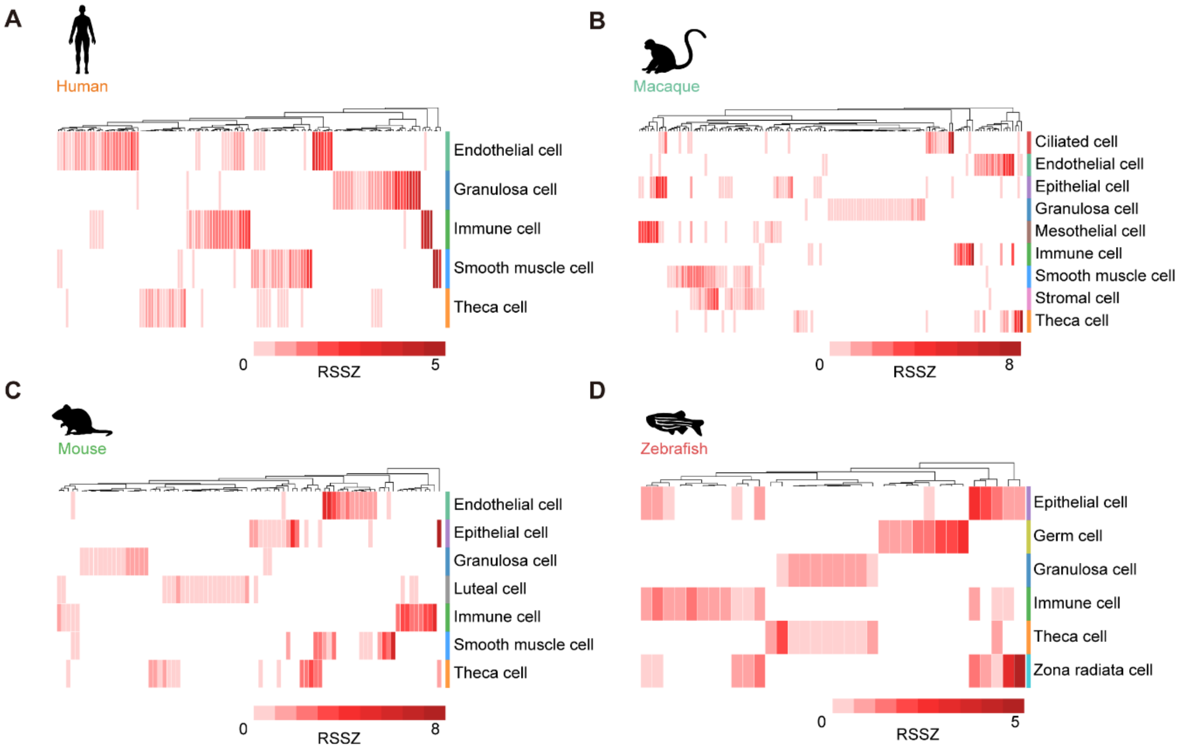

Supplementary Figure S6. Identification of cell type-specific transcription factors in vertebrate ovary (A) Heatmap showing the transcription factor Z-score normalized regulon specificity score (RSSZ) in different cell types of human ovary. (B) Heatmap showing the transcription factor RSSZ

in different cell types of macaque ovary. (C) Heatmap showing the transcription factor RSSZ in different cell types of mouse ovary. (D) Heatmap showing the transcription factor RSSZ in different cell types of zebrafish ovary.

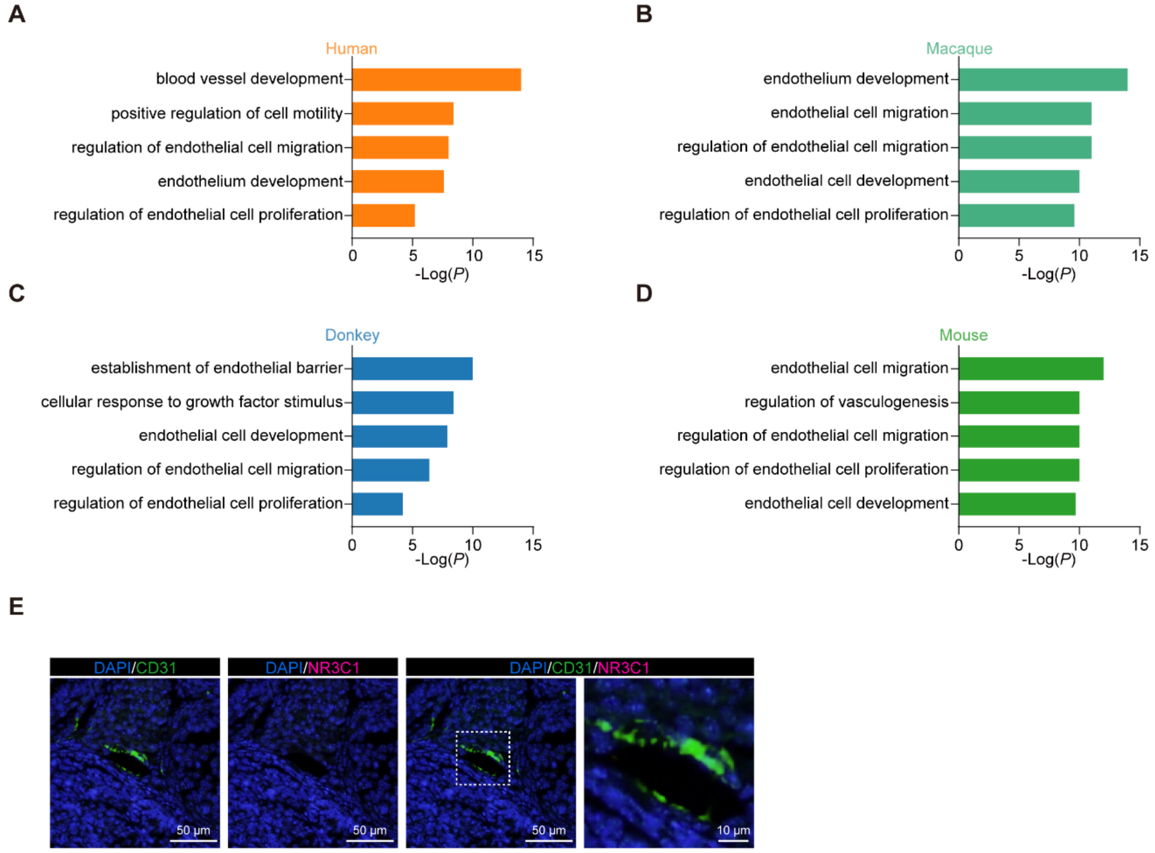

Supplementary Figure S7. Function and localization of transcription factors in mammalian ovarian endothelial cells (A) GO enrichment analysis results of *ETS1* target genes in human ovary. (B) GO enrichment analysis results of *ETS1* target genes in macaque ovary. (C) GO enrichment analysis results of *ETS1* target genes in donkey ovary. (D) GO enrichment analysis results of *ETS1* target genes in mouse ovary. (E) Representative images of NR3C1 staining (red) in mouse ovarian sections. Nucleus was counterstained with DAPI (blue). endothelial cells were labeled with CD31 (green).

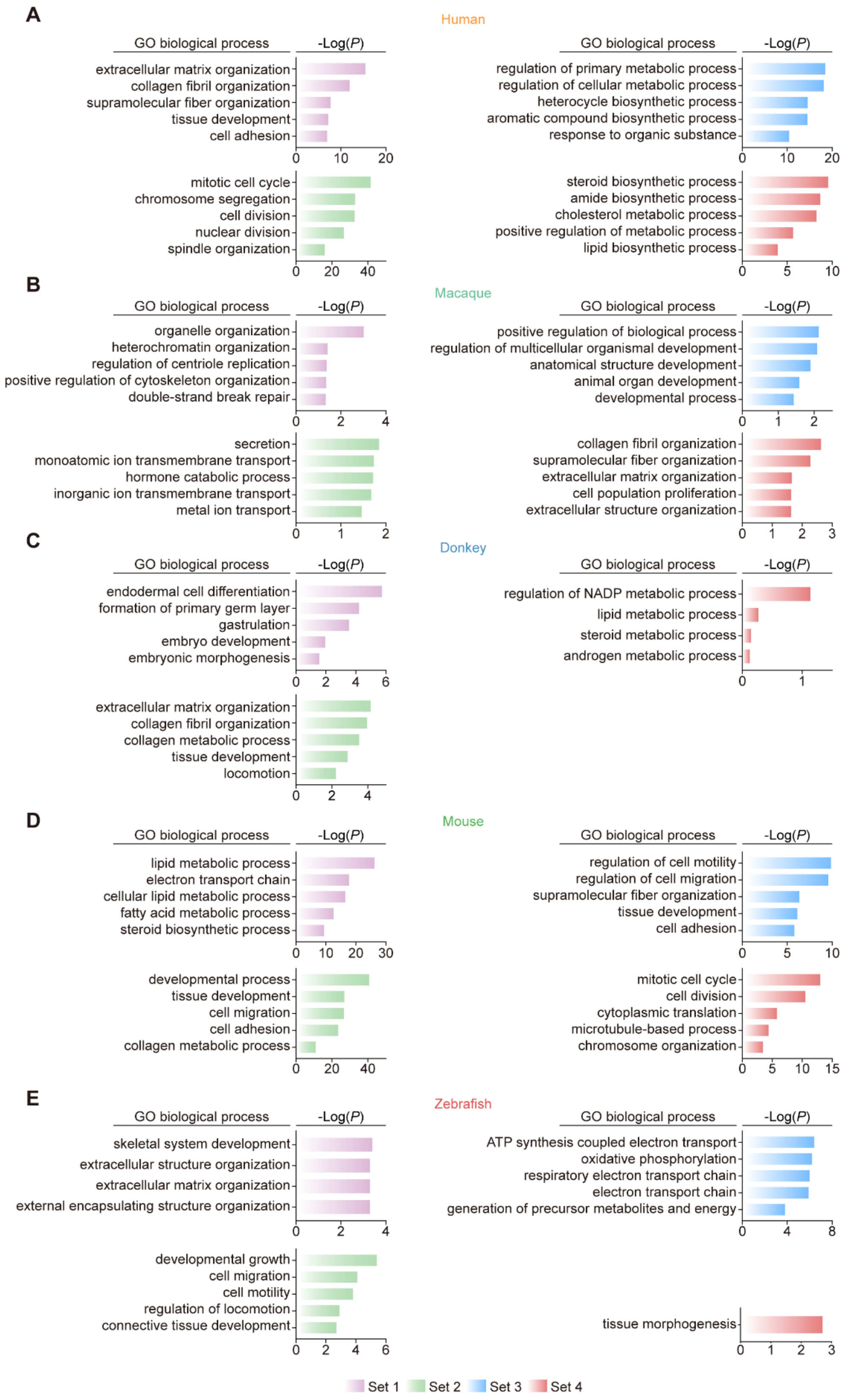

Supplementary Figure S8. Molecular characterization of theca cell subsets (A) GO terms associated with differentially expressed gene sets during human theca cell development. (B) GO terms associated with differentially expressed gene sets during macaque theca cell development. (C) GO terms associated with differentially expressed gene sets during donkey theca cell development. (D) GO terms associated with differentially expressed gene sets during mouse theca cell development. (E) GO terms associated with differentially expressed gene sets during zebrafish theca cell development.

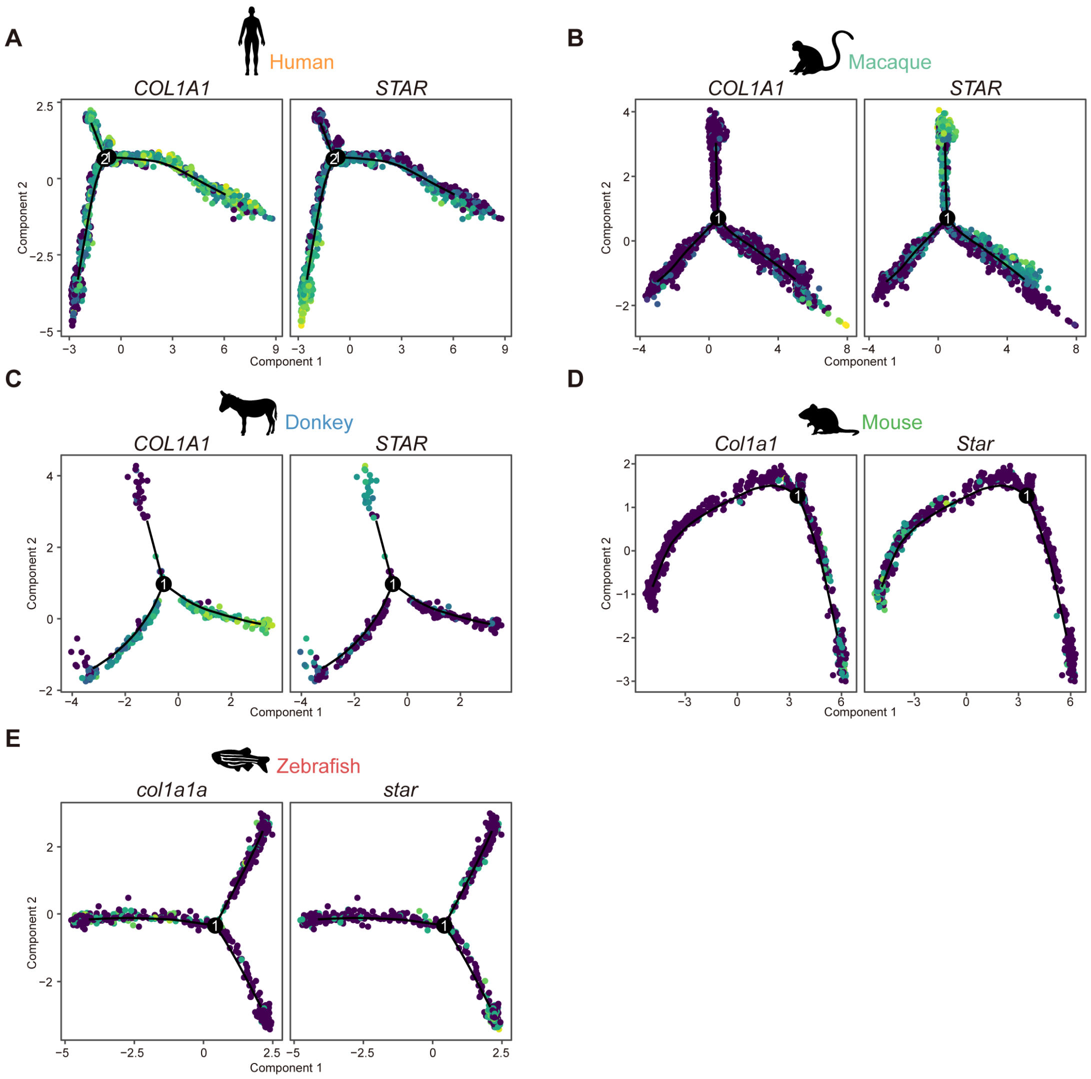

Supplementary Figure S9. Pseudotime developmental trajectories of vertebrate theca cells (A) Expression of *COL1A1* and *STAR* along with human theca cell pseudotime trajectories. (B) Expression of *COL1A1* and *STAR* along with macaque theca cell pseudotime trajectories. (C) Expression of *COL1A1* and *STAR* along with donkey theca cell pseudotime trajectories. (D) Expression of *Col1a1* and *Star* along with mouse theca cell pseudotime trajectories. (E) Expression of *col1a1a* and *star* along with zebrafish theca cell pseudotime trajectories.

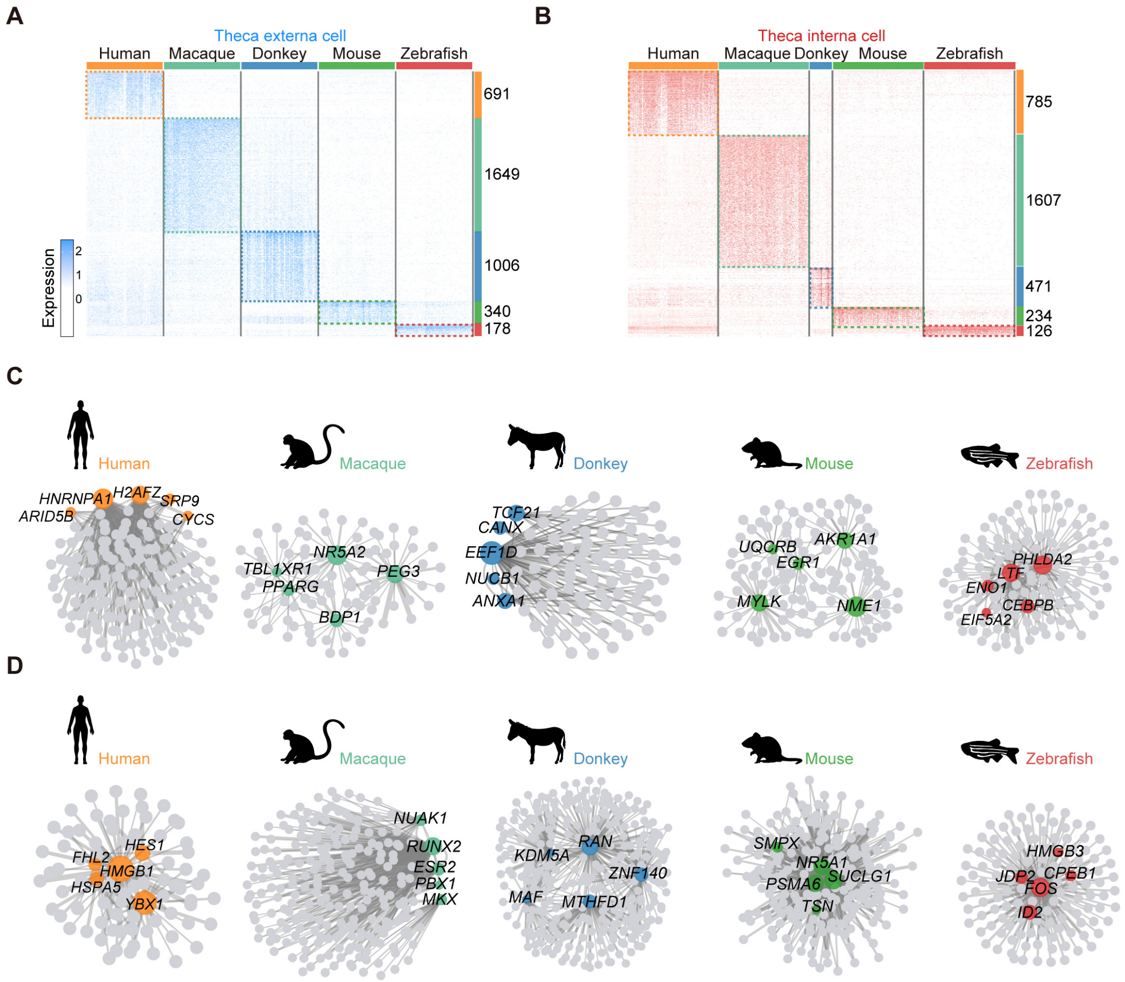

Supplementary Figure S10. Characteristics of theca cells of different species (A) Heatmap of up-regulated genes that are species-specific in theca externa cell. (B) Heatmap of up-regulated genes that are species-specific in theca interna cell. The numbers represent the number of genes specific to each species. (C) Regulatory networks visualizing potential key transcriptional regulators in species-specific upregulated genes in theca externa cell across different species. (D) Regulatory networks visualizing potential key transcriptional regulators in species-specific upregulated genes in theca interna cell across different species. Only connections with a high weight were retained. The line size indicates the weight of a connection. The node size represents the number of connections, with top-ranked transcription factors labeled by their gene names.

Table S1. Antibodies used in this paper

Primary antibodies	Vendor	Dilution	Source
CD31 (IF)	HUABIO (M1511-8)	1:200	Mouse
DHRS9 (IF)	Affinity (DF9438)	1:200	Rabbit
ETS1 (IF)	ABclonal (A15666)	1:100	Rabbit
GRHPR (IF)	ABclonal (A17593)	1:100	Rabbit
LIPE (IF)	Affinity (A24689)	1:200	Rabbit
NR3C1 (IF)	Affinity (AF5004)	1:100	Rabbit

Secondary antibodies	Vendor	Dilution	Source
Goat anti-mouse lgG H&L (Alexa Fluor® 488) (IF)	Abcam (ab150113)	1:200	Goat
Donkey anti-rabbit lgG H&L (Alexa Fluor® 555) (IF)	Abcam (ab150074)	1:200	Donkey

Table S2. Sequences of siRNA oligonucleotides

Gene	Forward primer sequence	Reverse primer sequence
SiGatm	GUCGAAGAGAUGUGCAAUATT	UAUUGCACAUCUCUUCGACTT
NC	UUCUCCGAACGUGUCACGUTT	ACGUGACACGUUCGGAGAATT

Table S3. Primers used for RT-qPCR

Gene	Forward primer sequence	Reverse primer sequence
Gapdh	GTCGGAGTGAACGGATTTGGC	CACCCCATTTGATGTTGGCG
Gatm	ACTAGGACCTTGTGCACGC	AAGGATCCTCCAAGCCGAGA
